# Carbohydrate-Based Chiral Iodoarene Catalysts: A Survey through the Development of an Improved Catalyst Design

**DOI:** 10.3390/molecules24213883

**Published:** 2019-10-28

**Authors:** Michael R. Imrich, Linda E. Biehler, Cäcilia Maichle-Mössmer, Thomas Ziegler

**Affiliations:** 1 Institute of Organic Chemistry, University of Tuebingen, Auf der Morgenstelle 18, 72076 Tuebingen, Germany; michael.imrich@uni-tuebingen.de (M.R.I.); linda.biehler@student.uni-tuebingen.de (L.E.B.); 2 Institute of Inorganic Chemistry, University of Tuebingen, Auf der Morgenstelle 18, 72076 Tuebingen, Germany; caecilia.maichle-moessmer@uni-tuebingen.de

**Keywords:** iodoarene, carbohydrate, dearomatization, spirolactonization, organocatalysis, asymmetric catalysis, Mitsunobu reaction, benzylic substitution, bulky substituent

## Abstract

Iodoarene catalysts can be applied in versatile reactions, for instance in the construction of complex chiral molecules via dearomatization of simple aromatic compounds. Recently, we reported the synthesis of the first carbohydrate-based chiral iodoarene catalysts and their application in asymmetric catalysis. Here we describe the synthesis of some new and improved catalysts. An account on how we got to the improved catalyst design, as well as the X-ray structure of one of the carbohydrate-based iodoarenes, is given.

## 1. Introduction

Carbohydrates are prevalent in nature. For instance, they occur as skeleton substances in plants or are used to store energy in almost all living creatures. Carbohydrates can be isolated from many different sources; therefore, common carbohydrates like d-glucose, d-galactose, or d-fructose are commercially available at low cost [1]. Bearing multiple stereo centers, carbohydrates contain a large amount of stereochemical information. Thus, they have already been widely used as starting materials for chiral auxiliaries as well as for the synthesis of chiral ligands for enantioselective, transition metal catalyzed reactions [2,3,4,5,6,7].

Transition metal catalyzed reactions provide a plethora of selective chemical modifications with a high tolerance against many functional groups [8]. Numerous catalytic reactions proceed in an enantioselective manner [9]. For a long time, metal-catalyzed reactions were nearly the only way of performing enantioselective catalytic reactions in an effective way. Since the beginning of the 2000s, the field of organocatalysis expanded rapidly, and many metal-free protocols for enantioselective catalysis can be found in the literature. Advantages of organocatalysis over transition metal catalysis are, for example, their lower toxicity and lower cost of the catalysts. Furthermore, organocatalysts are often more tolerant against moisture and oxygen [10].

**Scheme 1:**
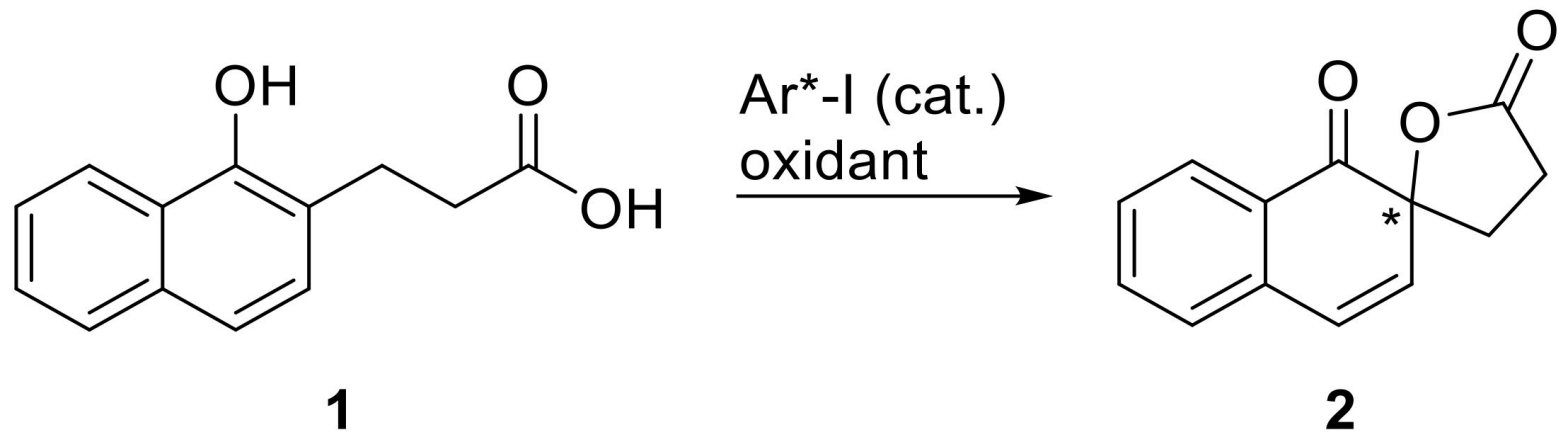
Enantioselective dearomatization of naphthole derivative **1** to **2** catalyzed by a chiral iodoarene.

**Figure 1:**
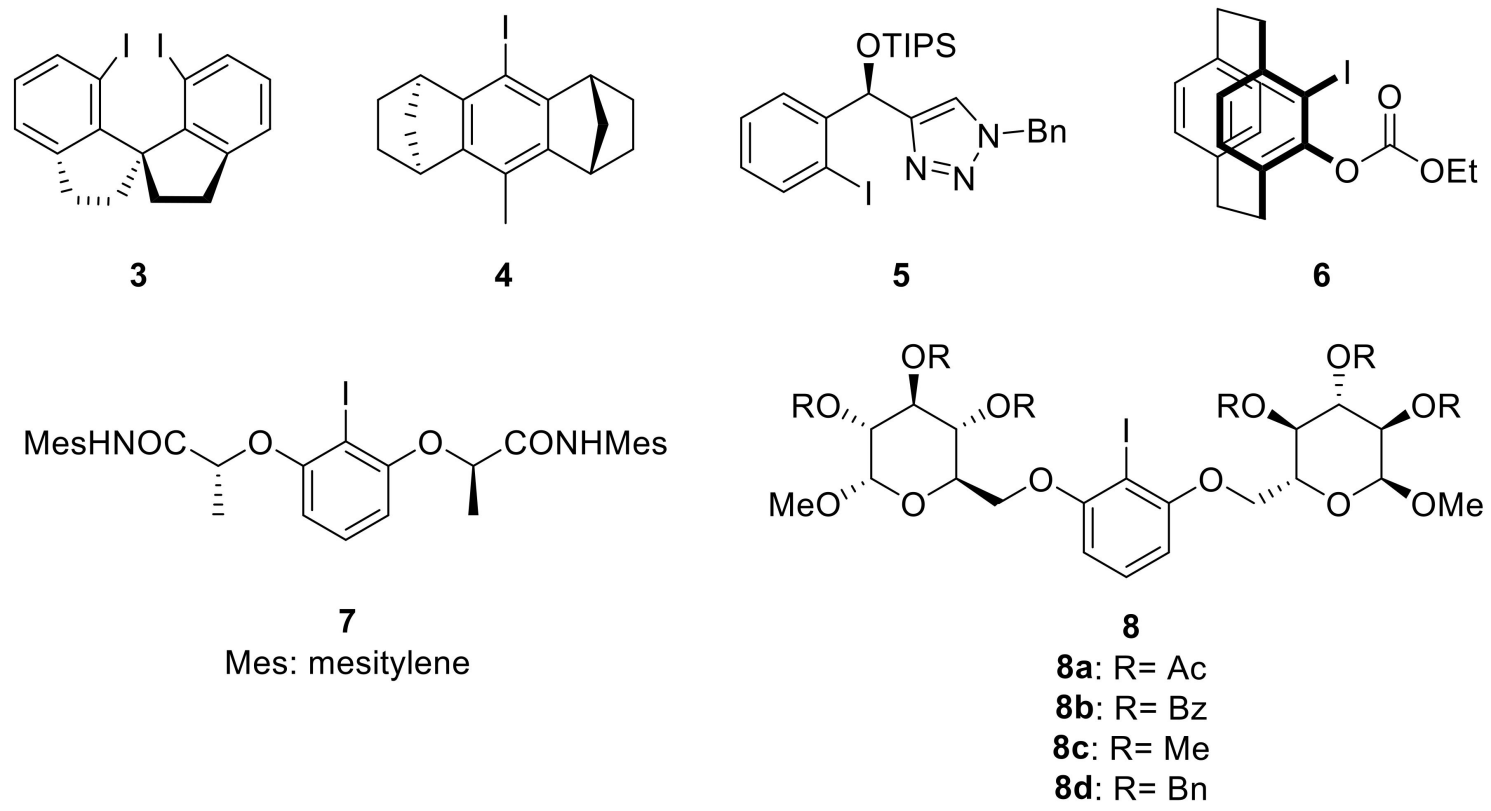
Some examples of chiral iodoarene catalysts described in the literature [15,16,17,18,19,20].

Chiral iodoarenes have gained significant importance as metal-free catalysts for stereoselective oxidation reactions. Oxidative dearomatization, for instance, provides the opportunity to construct complex molecules, like natural products, by starting from simple aromatic molecules [11,12,13]. Kita and coworkers developed a method for the enantioselective dearomatization of naphthole derivatives like compound **1** (Scheme 1) to the corresponding spiro lactone **2** using the chiral iodoarene catalyst **3** (Figure 1) and stoichiometric amounts of oxidant [14]. In the meantime, the iodoarene catalysts **4**–**7** (Figure 1) have been developed and were used in effective enantioselective dearomatizations of naphthole derivatives [15,16,17,18,19].

Recently, we reported on the synthesis of the first carbohydrate-derived chiral iodoarene catalysts **8** (Figure 1), which combined the benefits of carbohydrates as chiral auxiliary compounds with the advantages of organocatalysis for inexpensive, easily accessible, and robust catalysis processes. For example, catalyst **8d** gave an enantiomeric ratio (*er*) of **2** of 80:20 in 77% yield (Scheme 1) [20]. Here, we present a detailed study about the influence of the symmetric and stereochemical properties and the steric effects of the carbohydrate moiety of iodoarene catalysts in order to gain a better understanding of the origin of stereoinduction of these chiral catalysts.

## 2. Results and Discussion

**Scheme 2:**
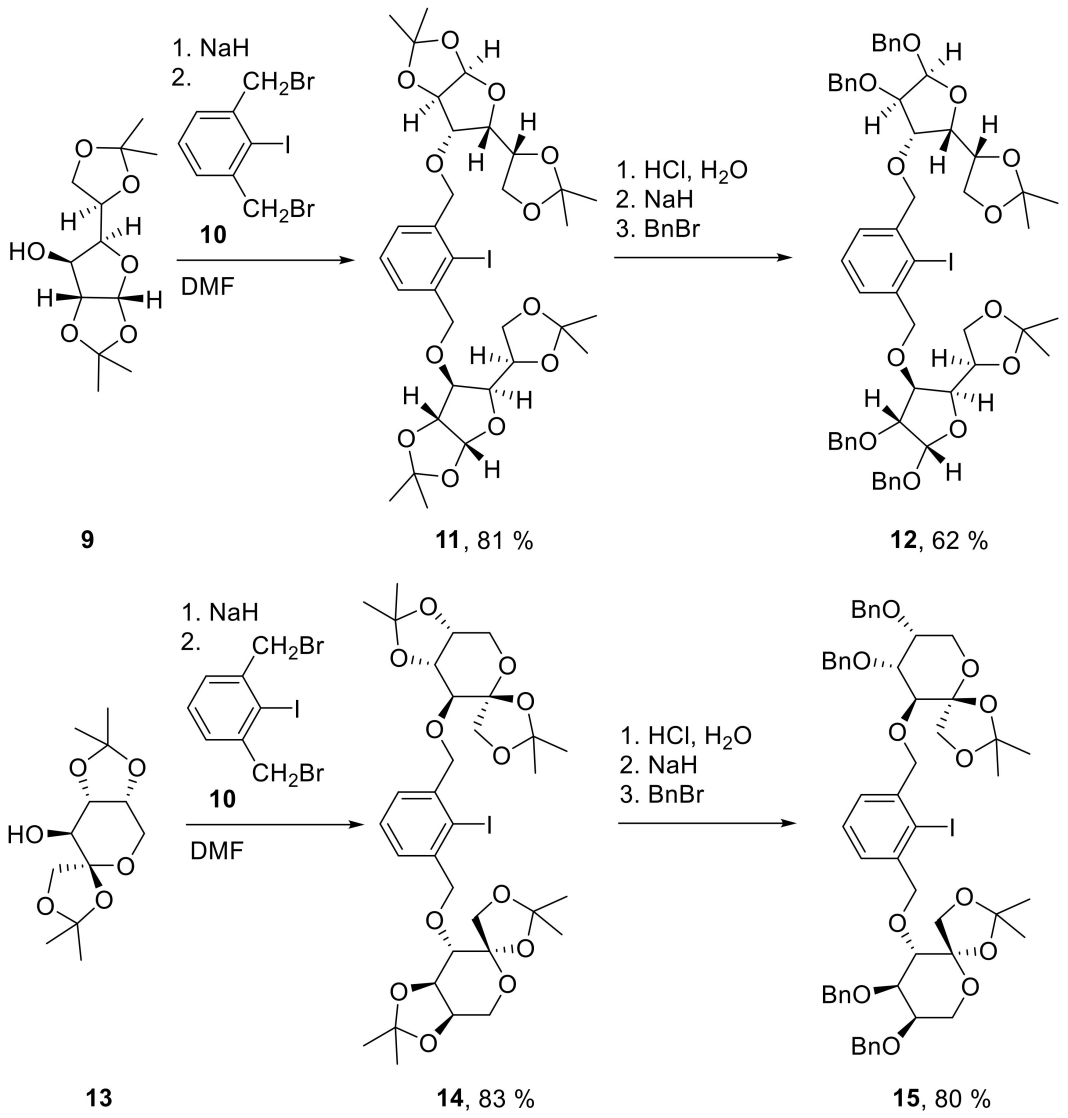
Preparation of catalysts **11**, **12**, **14**, and **15**.

Our first attempt to improve the enantioselectivity of our carbohydrate-based iodoarene catalysts was to change the linkage between the monosaccharide and the iodine-substituted aromatic ring. Instead of linking the carbohydrate moiety, via its position 6, and a phenyl ether, like in **8**, we now accomplished the linkage via a corresponding benzyl ether. An advantage of a benzylic connection of the sugar part over a phenolic connection is that the bond formation is not solely limited to position 6 at the monosaccharide, and other positions could be used as well. For this purpose, we chose 1,3-bis(bromomethyl)-2-iodo-benzene (**10**) as the iodoarene building block. The latter was prepared from 1,3-dimethyl-2-iodobenze according to a literature protocol [21]. For the introduction of the carbohydrate moiety, 1,2:5,6-di-*O*-isopropylidene-α-d-glucofuranose **9** and 1,2:4,5-di-*O* isopropylidene-β-d-fructopyranose **13** were prepared in one step from d-glucose and d-fructose, respectively, as was previously described [22,23]. Deprotonation of the hydroxyl group at position 3 in compounds **9** and **13** followed by subsequent addition of iodoarene **10** provided catalysts **11** and **14** in high yields (Scheme 2). We also changed the substitution pattern of **11** and **14** as follows. First, the isopropylidene groups at positions 5 and 6 in **11** and positions 4 and 5 in **14** were removed under acidic conditions, respectively. Next, the hydroxyl groups were benzylated using sodium hydride and benzyl bromide. Glucose-based catalyst **12** was, thus, isolated in 62% yield and the fructose-based catalyst **15** in 80% yield.

With four new carbohydrate-derived iodoarenes at hand, we next tested their potential as catalysts in enantioselective spirolactonization, for which we chose the identical conditions reported previously for catalyst **8** [20]. At −20 °C none of the new benzyl ether linked catalysts showed any conversion of **1** to **2** (Table 1, entries 2, 4, 6, and 8). At room temperature, catalysts **11** and **14** gave low yields of products (12% and 13%, respectively), though with a slight enrichment of the *R*-enantiomer in the product (Table 1, entries 3 and 5). Catalysts **12** and **15** were not at all capable of catalyzing the spirolactonization of **1**. Instead, a six-membered lactone between the naphtholic hydroxy and the acid group was formed (Table 1, entries 7 and 9). In comparison to the previously published phenol ether linked catalyst **8d**, yields as well as enantioselectivities were significantly lower for benzyl ether linked catalysts. 

**Scheme 3:**
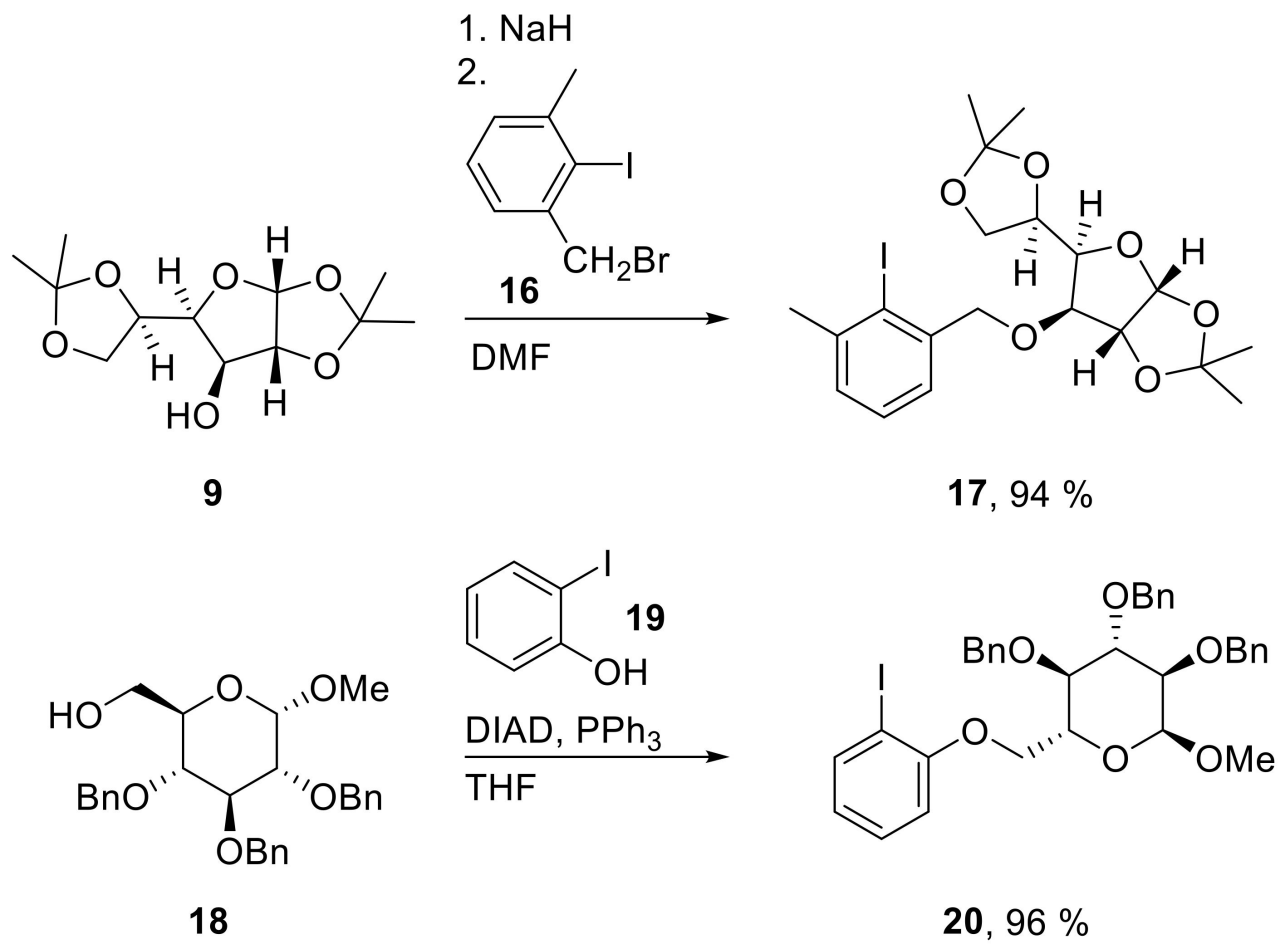
Synthesis of C_1_ symmetrical catalysts **17** and **20**.

Next, we tested whether decreasing the catalyst’s symmetry could lead to higher enantioselectivities because examples of C_1_ symmetric catalysts like **5** and **6** (Figure 1) are known to work well in asymmetric catalysis [16,19]. C_1_ symmetric catalyst **17** was prepared in high yield by deprotonation of **9** and subsequent addition of 1-(bromomethyl)-2-iodo-3-methylbenzene **16** (Scheme 3). The latter was obtained as a side product in the synthesis of **10.** For the preparation of catalyst **20**, a Mitsunobu protocol was used [24]. Condensation between commercially available 2-iodophenol **19** and known methyl d-glucoside **18** [25] afforded the respective carbohydrate-substituted iodoarene **20** in excellent yield. We selected **18** as the carbohydrate substituent for the catalyst because this substituent worked best in our previously reported catalyst **8**. 

Again, when **17** was applied as a catalyst in Kita’s spirolactonization, no conversion was observed at −20 °C (Table 1, entry 10). At room temperature, a small amount of **2** was isolated (Table 1, entry 11). Compared to the corresponding C_2_ symmetrical catalyst **11** (Table 1, entries 3 and 11), **20** also gave a slightly higher yield than **8d**, but the enantioselectivity was much lower in this case (Table 1, entries 1 and 12).

From those experiments, it can be generally concluded that C_2_ symmetric catalysts, having the sugar moiety linked to the iodoarene through a phenyl ether bond, gave better yields and higher enantioselectivities than C_1_ symmetric catalysts where the sugar was bound via a benzylic linkage. Therefore, we decided to focus next on catalysts having the same or similar structures as catalyst **8** but now varying the sugar and its anomeric configuration instead. 

**Scheme 4:**
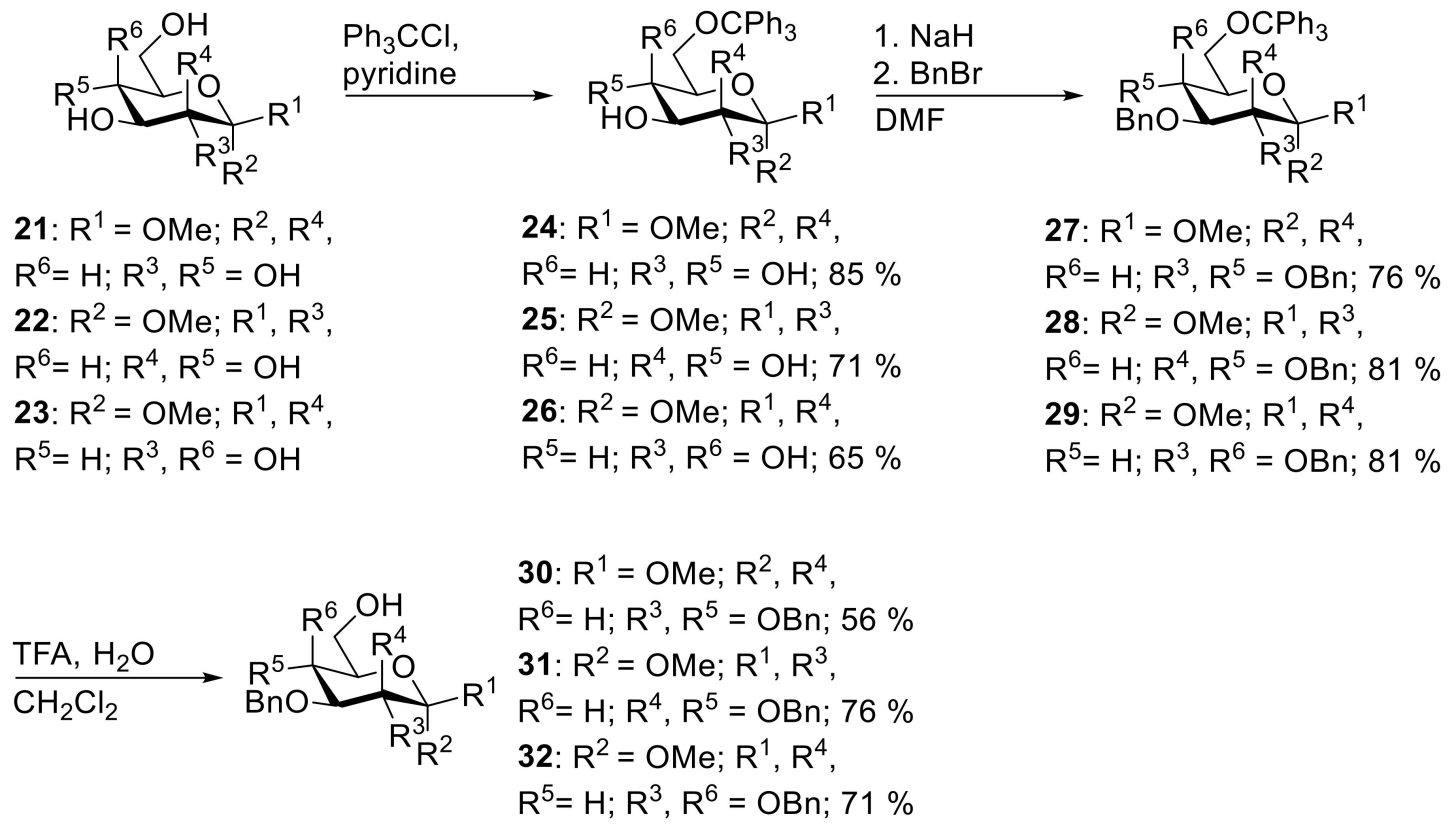
Synthesis of carbohydrate derivatives of different monosaccharides with similar substitution patterns at positions 1, 2, 3, and 4 and a hydroxyl group at position 6.

For the above-mentioned purpose, we prepared derivatives of methyl-β-d-glucopyranoside, methyl-α-d-mannopyranoside, and methyl-α-d-galactopyranoside with a similar substitution pattern like **18**. First, commercially available methyl glycosides **21**, **22**, and **23** were reacted with trityl chloride in pyridine to selectively protect the primary alcohol at position 6 (Scheme 4). Next, the remaining hydroxyl groups were benzylated using sodium hydride and benzyl bromide. Selective removal of the trityl group under acidic conditions gave catalyst precursors **30**, **31**, and **32**. 

**Scheme 5:**
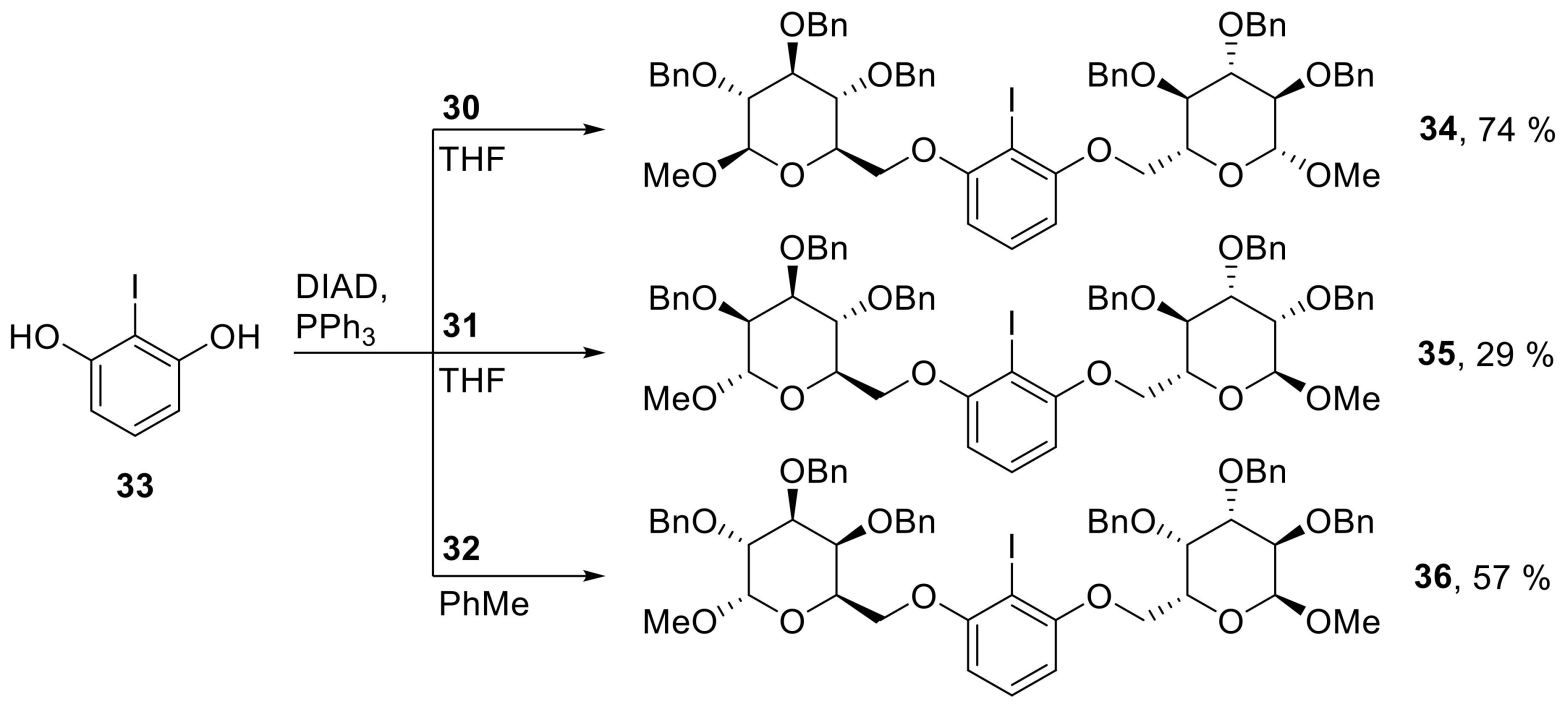
Mitsunobu reaction between 2-iodoresorcinol **33** and carbohydrate derivatives **30**, **31**, and **32** to synthesize catalysts **34**, **35**, and **36**.

Again we used a Mitsunobu protocol for the condensation of 2-iodoresorcinol (**33**) [24] and carbohydrate derivatives **30**, **31**, and **32** (Scheme 5). In the case of glucose- and mannose-based precursors, the previously used method (DIAD, PPh_3_, THF, room temperature) worked well. Carbohydrate-substituted iodoarenes **34** and **35** were obtained in 74% and 29% yields, respectively. In the case of galactose derivative **32**, however, an inseparable mixture of products was formed. After slightly changing the Mitsunobu protocol (DIAD, PPh_3_, PhMe, reflux), as was previously described in similar cases [26], galactose-based iodoarene **36** was isolated in fair yield (57%) (Scheme 5). 

The substituted iodoarenes **34**, **35,** and **36** were then applied as catalysts in Kita’s spirolactonization of 1-hydroxy-2-naphthalenepropionic acid (**1**). β-Configurated **34** gave 73% yield and an enantiomeric ratio of 70:30 (Table 1, entry 13). Compared to catalyst **8d**, which had an α-configuration at the anomeric center, the yield was in a similar range, and the enantioselectivity was only slightly lower. Mannose-derived catalyst **35** had an axial substituent at position 2, whereas the respective substituent in **8d** was equatorial. Catalyst **35** gave a lower yield and slightly lower enantioselectivity when applied in Kita’s spirolactonization (Table 1, entry 14) than **8d** (Table 1, entry 1). Galactose-derived catalyst **36** had an inverted configuration at position 4 compared to **8d**. The yield when using **36** as a catalyst was in a similar range as that of **35**. However, the enantiomeric ratio decreased significantly. The almost complete loss of stereoinduction by changing the configuration at position 4 led us to the assumption that this stereocenter is of major importance for the catalyst design. 

**Figure 2:**
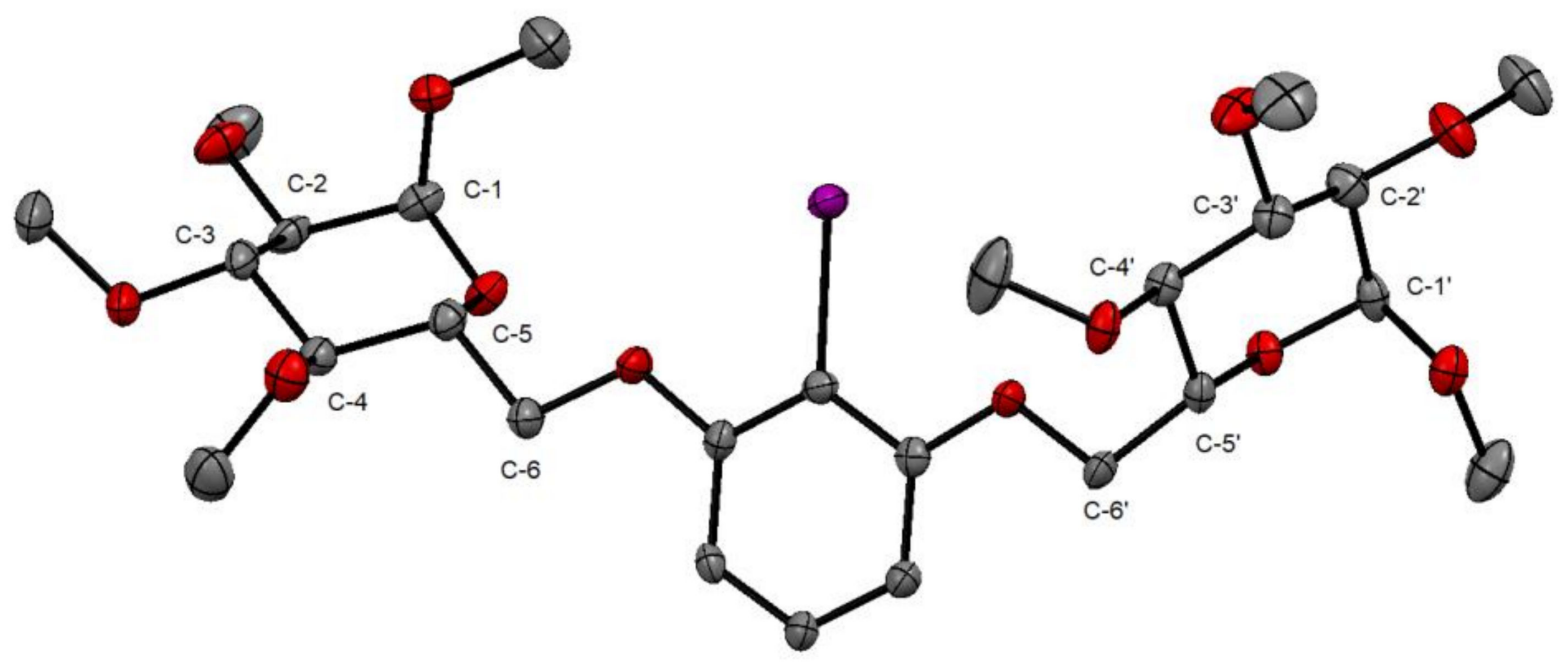
Molecular structure of **8c**. Ellipsoids are given at the 50% probability level; hydrogens are omitted for better clarity. Grey: carbon, red: oxygen, purple: iodine.

In order to verify our assumption of the importance of position 4 of the carbohydrate moiety further, we first took a closer look at the molecular structure of the C_2_ symmetrical phenyl ether linked catalysts. For that purpose, we tried to crystallize catalyst **8** as well as **34**, **35,** and **36**. However, we could not get useful crystallographic data on catalysts **8a**, **8b**, **8d**, **34**, **35**, and **36**. Fortunately, we were able to obtain crystals of compound **8c** that were suitable for X-ray crystallography. Compound **8c** bears methyl groups instead of benzyl groups at positions 2, 3, and 4 like **8d** does. Compound **8c** gave an *er* of 69:31 and 61% yield by its application in Kitas spirolactonization, which was slightly lower than **8d** (78:22; 67% at the same reaction conditions) [20]. Despite the fact that different protective groups might cause changes in the conformation of the catalyst, the X-ray structure of **8c** can be used as a model system to get a better idea of the molecular structure of our catalysts. The molecular structure of **8c** is shown in Figure 2. The molecule crystallized in the orthorhombic space group *P*2_1_2_1_2_1_ [27]. The molecular structure showed that the substituents at C-4 and C-4′ were both located close to the iodine where the catalytic reaction took place. The substituents at the anomeric centers did not come as close as the substituents at position 4, and the groups at positions 2 and 3 even pointed in different directions. Thus, we assumed that a very bulky substituent at position 4 was be beneficial for the enantioselectivity and that the other substituents were not that important. 

In order to investigate the influence of the substituent at position 4 in greater detail, catalysts with very bulky substituents at this position and “small” substituents (methyl groups) at positions 1, 2, and 3 were prepared. For the substituents at position 4 we chose 3,5-dimethyl-benzyl, 3,5-di-*tert*-butyl-4-methoxy-benzyl and 1-naphthylmethyl since they were sufficiently bulky, were stable under the reaction conditions, and also could be easily introduced by using the corresponding benzylic bromides. The latter bromides were either commercially available or were prepared as described previously [3]. 

**Scheme 6:**
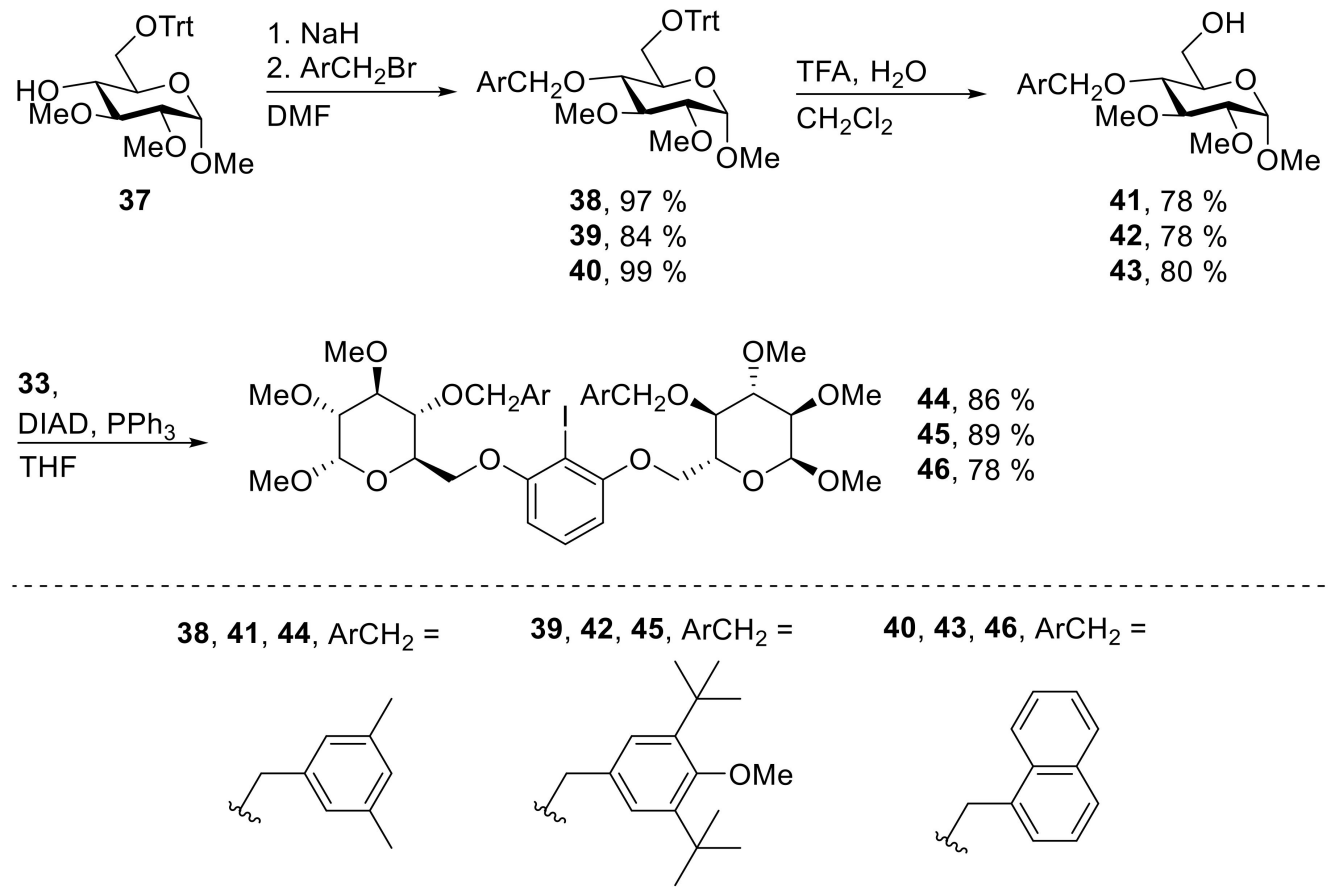
Preparation of α-d-glucose-based iodoarenes **44**, **45**, and **46** with a bulky substituent at position 4 and small methyl groups at positions 1, 2, and 3.

We started the preparation of the known 1,2,3-trimethyl-6-trity-glucose (**37**) [28]. Reaction of the latter with different benzyl bromides under basic conditions afforded glycosides **38**, **39**, and **40** in good to virtually quantitative yields (Scheme 6). Removal of the trityl protective group at position 6 with trifluoroacetic acid afforded primary alcohols **41**, **42**, and **43** in good yields as well. Finally, alcohols **41**−**43** were coupled with 2-iodoresorcinol (**33**), using DIAD and PPh_3_ as reagents, to afford carbohydrate-substituted iodoarenes **44**, **45**, and **46**. Catalysts **44**, **45**, and **46** were than applied in Kita’s spirolactonization of naphthole derivative **1**. The catalysts with the substituted benzyl groups at position 4 gave similar enantiomeric ratios (79:21 for **44** and 81:19 for **45** respectively) and yields ranging between 70% and 80% (Table 1, entries 16 and 17). Enantioselectivities and yields were in a similar range as for the previously published catalyst **8d**. 1-Naphthylmethyl-substituted α-d-glucose-based iodoarene **46**, however, resulted in a yield in the same range as for **44** and **45** but in a slightly higher enantioselectivity (*er* 86:14, Table 1, entry 18), which proved our assumption that the substituents at position 4 of the sugar moiety were critical.

Finally, we also prepared a catalyst with sterically demanding 1-naphthylmethyl groups at all positions of the sugar moieties. If position 4 is indeed the main origin of stereoinduction in our catalyst design, such fully 1-naphthylmethyl protected iodoarenes should provide similar selectivities like **46**.

**Scheme 7:**
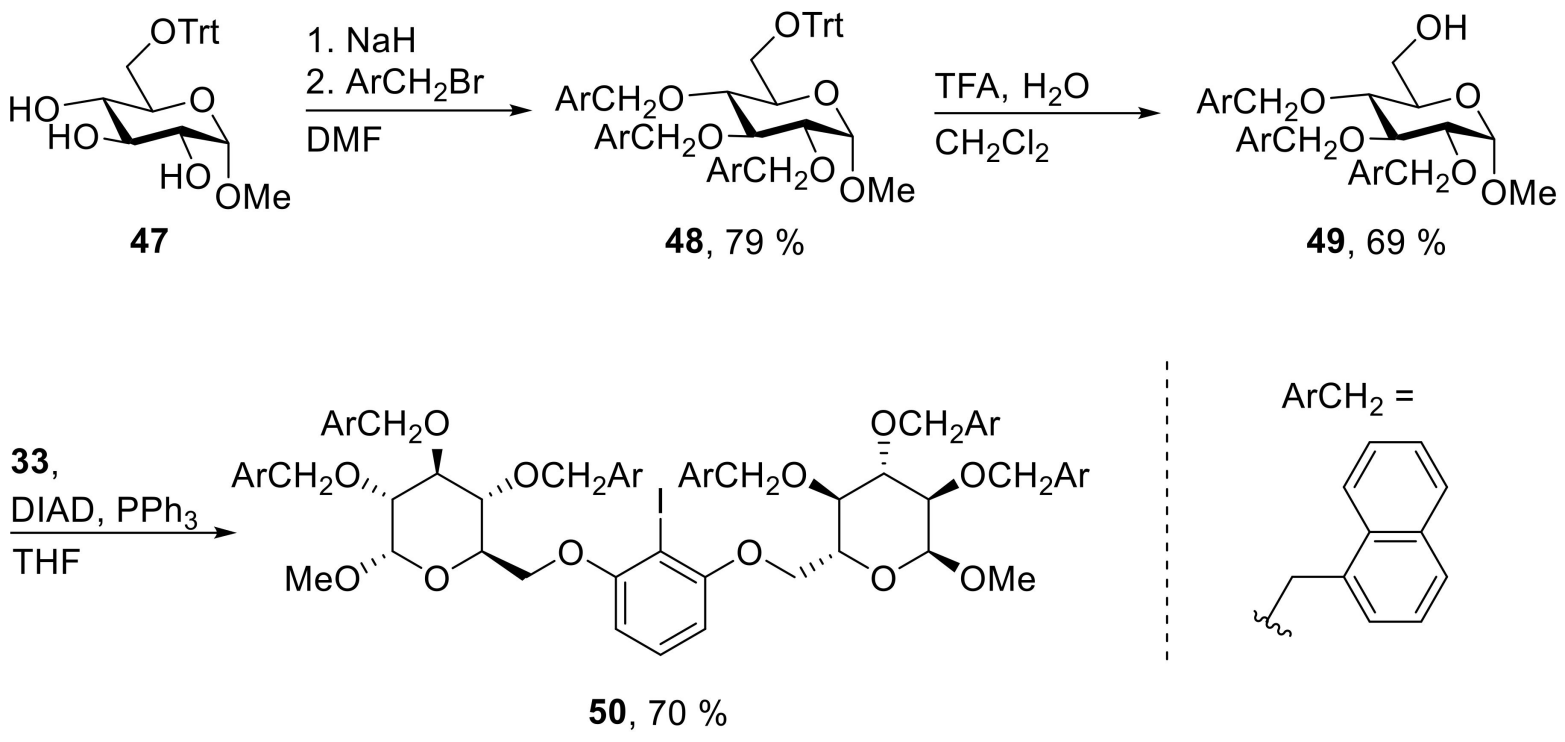
Synthesis of 1-naphthylmethyl-substituted catalyst **50**.

Once again, the preparation (Scheme 7) started with known 1-methyl-6-trityl-α-d-glucose **47** [25], which was reacted with 1-(bromomethyl)naphthalene to give glucoside **48** in 79% yield. Selective deprotection of the trityl group in **48**, followed by Mitsunobu esterification of intermediate **49** with 2-iodoresocinol **33**, afforded iodoarene **50** in 70% yield. The application of **50** in Kita’s spirolactonization resulted in 75% yield, which was nearly identical to the yield obtained with catalyst **46** (Table 1, entry 19). The enantiomeric ratio was slightly higher than with **46** (**50**: 88:12, **46:** 86:14), but it was still in a similar range. 

Indeed, position 4 at the monosaccharide derivative is most important for the stereoinduction of our carbohydrate-based iodoarene catalyst, but the other positions seem to have some influence too.

## 3. Materials and Methods

### 3.1. General Remarks

Reactions in dry solvents were carried out under an atmosphere of nitrogen using Schlenk techniques. Dry THF and PhMe were distilled from sodium and benzophenone, and dry CH_2_Cl_2_ and DMF were distilled from P_4_O_10_. Solvents used for preparative column chromatography were of technical grade and distilled prior to their use. Petroleum ether (PE) refers to the fraction boiling at 60−90 °C. Silica gel “60 M” from Macherey-Nagel (Düren, Germany) was used for preparative column chromatography. For TLC, “Polygram Sil G/U_254_” plates purchased from Machery-Nagel were used. Melting points were measured with a Büchi “Melting Point M-560” apparatus (Flawil, Switzerland). Optical rotations were determined with a Perkin-Elmer “Polarimeter 341” (Wattham, MA, USA). NMR spectra were measured with a Bruker “Avance III HD 400” or a Bruker “Avance III HD 300 NanoBay” spectrometer (Billerica, MA, USA). NMR spectra were calibrated to the solvent signal (CDCl_3_: ^1^H 7.27 ppm, ^13^C 77.0 ppm; CD_2_Cl_2_^1^H 5.32 ppm, ^13^C 53.8 ppm; PhMe-d_8_: ^1^H 2.08 ppm, ^13^C 20.81 ppm). Additional spectra (DEPT-135; ^1^H,^1^H-COSY; ^1^H,^13^C-HMBC; ^1^H,^13^C-HSQC) were recorded for peak assignment, and the atoms were numbered according to the carbohydrate nomenclature. Chemical shifts were given in ppm. High-resolution mass spectra (HRMS) were recorded on a Bruker “maXis 4G” with electrospray ionization and a time-of-flight detector. Elemental analysis was performed using a HEKAtech “Euro 3000 CHN” (Wegberg, Germany). Enantiomeric ratios were determined by chiral HPLC using a Dr Maisch (Ammerbuch-Entringen, Germany) “Reprosil Chiral-OM, 5 μm, 125 × 4.6 mm” column with *n*-hexanes/2-propanol, 85/15, as eluent and 0.7 mL/min as the flow rate. The Supplementary Materials contains copies of NMR spectra, HPLC chromatograms, and crystallographic data.

### 3.2. Experimental Procedures

#### 3.2.1. General Procedure A: Benzylic Substitution 

Respective carbohydrate derivative (1.0 equiv) was dissolved in dry DMF (0.15 m), and NaH (2.0 equiv for each hydroxyl group; 60% dispersion in mineral oil) was added in small portions. After complete addition, the resulting mixture was stirred for 10 min at room temperature. The reaction mixture was cooled in an ice bath, and the particular benzylic bromide (1.0 to 1.5 equiv for each hydroxyl group) was slowly added. The mixture was allowed to reach room temperature and was stirred until TLC showed complete consumption of the starting material, at which point the reaction was quenched by the addition of MeOH. The solvent was evaporated in vacuo, and the residue was re-dissolved in H_2_O and EtOAc. The aqueous layer was extracted with EtOAc (3 times), and the combined organic layers were dried (Na_2_SO_4_). The crude product was purified by column chromatography.

#### 3.2.2. General Procedure B: Changing Isoproylidene Groups to Benzyl Groups

The respective isopropylidene-protected compound (1.0 equiv) was dissolved in THF (0.03 m), aqueous HCl (10 mL for each mol of isopropylidene-protected compound; 2 m) was added, and the resulting mixture was stirred at room temperature until TLC indicated complete consumption of the starting material. The reaction mixture was neutralized with solid NaHCO_3_, and the solvent was evaporated in vacuo. The solid residue was extracted with 2-propanol, which was evaporated in vacuo afterwards. The crude product was dissolved in dry DMF (0.05 m with respect to the isopropylidene-protected compound), and NaH (4.0 equiv for each removed isopropylidene group, 60% dispersion in mineral oil) was added. The reaction mixture was stirred 10 min at room temperature, and BnBr (3.0 equiv for each removed isopropylidene group) was added slowly. The reaction mixture was stirred at room temperature overnight, quenched with MeOH, and the solvent evaporated in vacuo. The residue was re-dissolved in H_2_O and EtOAc, the aqueous layer was extracted with EtOAc (3 times), and the combined organic layers were dried (Na_2_SO_4_). The crude product was purified by column chromatography.

#### 3.2.3. General Procedure C: Mitsunobu Reaction 

Particular iodophenol (1.0 equiv) and carbohydrate derivatives (1.0 to 1.1 equiv for each hydroxyl group at the iodophenol) were dissolved in dry THF (0.2 m with respect to the iodophenol), and PPh_3_ (1.15 equiv for each hydroxyl group at the iodophenol) and DIAD (1.2 equiv for each hydroxyl group at the iodophenol) were added. The resulting mixture was stirred at room temperature for 15 h, PPh_3_ (0.3 equiv for each hydroxyl group at the iodophenol) and DIAD (0.3 equiv for each hydroxyl group at the iodophenol) were added, and the reaction mixture was stirred at room temperature for an additional 4 h. The solvent was evaporated in vacuo, and the residue was purified by column chromatography.

#### 3.2.4. General Procedure D: Mitsunobu Reaction 

Compound **33** (1.0 equiv) and PPh_3_ (3.0 equiv) were dissolved in dry PhMe (0.05 m with respect to **33**), DIAD (3.0 equiv) was added, and the mixture was stirred at room temperature for 5 min. A solution of carbohydrate derivative (2.5 equiv) in dry PhMe (0.2 m) was added, and the reaction mixture was heated to reflux for 17 h. The solvent was evaporated in vacuo, and the residue was purified by column chromatography.

#### 3.2.5. General Procedure E: Introduction of the Trityl Group

A solution of methyl glycoside (1.0 equiv) and Ph_3_CCl (1.2 equiv) in dry pyridine was heated to 60 °C until TLC showed complete consumption of the starting material. MeOH was added, and the solvent was evaporated in vacuo. The residue was coevaporated with PhMe (3 times) and purified by column chromatography.

#### 3.2.6. General Procedure F: Cleavage of the Trityl Group

To a solution of trityl-protected compound (1 equiv) in CH_2_Cl_2_ (0.3 m), H_2_O (4 equiv) and TFA (2 equiv) were added, and the mixture was stirred at room temperature until TLC showed complete consumption of the starting material. The reaction mixture was diluted with CH_2_Cl_2_ and H_2_O. The aqueous phase was saturated with NaCl and extracted with CH_2_Cl_2_ (3 times). The combined organic layers were washed with saturated NaHCO_3_ solution and dried (Na_2_SO_4_). The crude product was purified by column chromatography.

#### 3.2.7. General Procedure G: Kita’s Spirolactonization

The spirolactonization was performed according to known procedure in the literature, and the enantiomeric ratio was determined as described previously [16,20].

### 3.3. Characterization of Compounds

**11**: Prepared from **9** (599 mg, 2.30 mmol) and **10** (390 mg, 1.00 mmol) accordingly to general procedure A; colorless solid; 81% (604 mg) yield after column chromatography (PE/EtOAc, 5/1); R_f_ = 0.53 (PE/EtOAc, 5/1); mp = 56 °C (PE, EtOAc); [α]^20^_D_ = −23.3° (c = 1.0, CHCl_3_); ^1^H-NMR (300 MHz, CDCl_3_) δ = 7.29–7.47 (m, 3H, H-Ar), 5.93 (d, *J* = 3.8 Hz, 2H, H-1), 4.63–4.81 (m, 6H, CH_2_Ar, H-2), 4.35–4.45 (m, 2H, H-5), 4.08–4.21 (m, 6H, H-3, H-4, H-6b), 3.98–4.04 (m, 2H, H-6a), 1.52 (s, 6H, CH_3_), 1.44 (s, 6H, CH_3_), 1.35 (s, 6H, CH_3_), 1.35 (s, 6H, CH_3_); ^13^C-NMR (75 MHz, CDCl_3_) δ = 140.4, 128.1, 128.0 (C-Ar), 111.9, 109.1 (*C*(CH_3_)_2_), 105.3 (C-1), 100.9 (CI), 82.5 (C-2), 82.2 (C-5), 81.3 (C-3), 76.6 (CH_2_Ar), 72.4 (C-4), 67.5 (C-6), 26.8, 26.8, 26.3, 25.5 (CH_3_); HRMS (ESI-TOF) *m/z* [M + Na]^+^: calcd for C_32_H_45_IO_12_Na: 771.18480, found: 771.18465; Anal calcd for C_32_H_45_IO_12_: C 51.34, H 6.06, found: C 51.37, H 6.20.

**12**: Prepared from **11** (408 mg, 0.545 mmol) accordingly to general procedure B; colorless syrup; 62% (348 mg) yield after column chromatography (PE/EtOAc, 5/1 → 3/1); R_f_ = 0.41 (PE/EtOAc, 3/1); [α]^20^_D_ = −34.3° (c = 1.0, CH_2_Cl_2_); ^1^H-NMR (400 MHz, CD_2_Cl_2_) δ = 7.15–7.41 (m, 23H, H-Ar), 5.89 (d, *J* = 3.8 Hz, 2H, H-1), 4.64–4.79 (m, 6H, CH_2_Ar, H-2), 4.57 (s, 4H, CH_2_Ar), 4.44 (dd, *J* = 8.5, 11.9 Hz, 4H, CH_2_Ar), 4.30 (dd, *J* = 2.9, 9.2 Hz, 2H, H-4), 4.16 (d, *J* = 2.9 Hz, 2H, H-3), 3.97–4.04 (m, 2H, H-5), 3.91 (dd, *J* = 1.8, 10.6 Hz, 2H, H-6a), 3.67 (dd, *J* = 5.4, 10.6 Hz, 2H, H-6b), 1.48 (s, 6H, CH_3_), 1.31 (s, 6H, CH_3_); ^13^C-NMR (101 MHz, CD_2_Cl_2_) δ = 141.2, 139.4, 139.3, 128.8, 128.7, 128.4, 128.1, 128.0, 128.0, 127.9 (C-Ar), 112.3 (*C*(CH_3_)_2_), 105.8 (C-1), 101.1 (CI), 82.7 (C-3), 82.2 (C-2), 79.5 (C-4), 76.8 (CH_2_Ar), 76.3 (C-5), 73.9, 72.9 (CH_2_Ar), 71.5 (C-6), 27.1, 26.6 (CH_3_); HRMS (ESI-TOF) *m/z* [M + Na]^+^: calcd for C_54_H_61_IO_12_Na: 1051.31000, found: 1051.31032; Anal calcd for C_54_H_61_IO_12_: C 63.03, H 5.98, found: C 63.31, H 6.12.

**14**: Prepared from **13** (599 mg, 2.30 mmol) and **10** (390 mg, 1.00 mmol) accordingly to general procedure A; colorless solid; 83% (620 mg) yield after column chromatography (PE/EtOAc, 4/1); R_f_ = 0.59 (PE/EtOAc, 2/1); mp = 148 °C (PE, EtOAc); [α]^20^_D_ = −79.3° (c = 1.0, CHCl_3_); ^1^H-NMR (400 MHz, CDCl_3_) δ = 7.40–7.48 (m, 2H, H-Ar), 7.26–7.34 (m, 1H, H-Ar), 4.99–5.08 (m, 2H, CH_2_Ar), 4.63–4.74 (m, 2H, CH_2_Ar), 4.39 – 4.44 (m, 2H, H-4), 4.25 (dd, *J* = 2.1, 5.6 Hz, 2H, H-5), 4.12–4.19 (m, 4H, H-1b, H6-b), 3.99–4.07 (m, 2H, H-6a), 3.93 (d, *J* = 8.6 Hz, 2H, H-1a), 3.59–3.65 (m, 2H, H-3), 1.61 (s, 6H, CH_3_), 1.51 (s, 6H, CH_3_), 1.44(s, 6H, CH_3_), 1.46 (s, 6H, CH_3_); ^13^C-NMR (101 MHz, CDCl_3_) δ = 141.1, 128.3, 127.9, (C-Ar), 112.2, 109.1 (*C*(CH_3_)_2_), 104.4 (C-2), 101.1 (CI), 77.6 (C-4), 76.9 (C-3), 73.9 (C-5), 72.9 (CH_2_Ar), 72.0 (C-1), 60.2 (C-6), 28.2, 26.9, 26.3, 26.1 (CH_3_); HRMS (ESI-TOF) *m/z* [M + Na]^+^: calcd for C_32_H_45_IO_12_Na: 771.18480, found: 771.18507; Anal calcd for C_32_H_45_IO_12_: C 51.34, H 6.06, found: C 51.45, H 6.12.

**15**: Prepared from **14** (363 mg, 0.485 mmol) accordingly to general procedure B; colorless solid; 80% (400 mg) yield after column chromatography (PE/EtOAc, 4/1); R_f_ = 0.27 (PE/EtOAc, 3/1); mp = 54 °C (*n*-heptane, CH_2_Cl_2_); [α]^20^_D_ = −61.7° (c = 1.0, CH_2_Cl_2_); ^1^H-NMR (400 MHz, CD_2_Cl_2_) δ = 7.22–7.51 (m, 23H, H-Ar), 5.07 (d, *J* = 13.0 Hz, 2H, H-1a), 4.60–4.75 (m, 8H, H-1b, CH_2_Ar), 4.55 (d, *J* = 11.5 Hz, 2H, CH_2_Ar), 4.08 (d, *J* = 8.4 Hz, 2H, CH_2_Ar), 3.76–4.02 (m, 12H, CH_2_Ar, H-3, H-4, H-5, H-6a, H-6b), 1.48 (s, 6H, CH_3_), 1.43 (m, 6H, CH_3_); ^13^C-NMR (101 MHz, CD_2_Cl_2_) δ = 141.9, 139.1, 139.0, 128.9, 128.8, 128.5, 128.4, 128.3, 128.2, 128.2, 128.0, 127.9 (C-Ar), 112.3 (*C*(CH_3_)_2_), 106.4 (C-2), 100.1 (CI), 80.5(C-5), 79.9 (C-1), 76.0 (C-3), 74.5 (C-4), 72.6, 72.3, 72.1 (CH_2_Ar), 61.8 (C-6), 27.4, 26.8 (CH_3_); HRMS (ESI-TOF) *m/z* [M + Na]^+^: calcd for C_54_H_61_IO_12_Na: 1051.31000, found: 1051.30988, Anal calcd for C_54_H_61_IO_12_: C 63.03, H 5.98; found: C 63.07, H 6.09.

**17**: Prepared from **9** (312 mg, 1.20 mmol) and **16** (311 mg, 1.00 mmol) accordingly to general procedure A; slight yellow oil; 94% (460 mg) yield after column chromatography (PE/EtOAc, 5/1); R_f_ = 0.66 (PE/EtOAc, 2/1); [α]^20^_D_ = −13.0° (c = 1.0, CHCl_3_); ^1^H-NMR (400 MHz, CDCl_3_) δ = 7.21–7.35 (m, 3H, H-Ar), 5.97 (d, *J* = 3.7 Hz, 1H, H-1), 4.67–4.81 (m, 3H, H-2, CH_2_), 4.42–4.48 (m, 1H, H-5), 4.13–4.24 (m, 3H, H-4, H-6b), 4.05 (dd, *J* = 6.0, 8.4 Hz, 1H, H-6a), 2.52 (s, 3H, CH_3_Ar), 1.56 (s, 3H, CH_3_), 1.48 (s, 3H, CH_3_), 1.40 (s, 3H, CH_3_), 1.38 (s, 3H, CH_3_); ^13^C-NMR (101 MHz, CDCl_3_) δ = 142.1, 140.5, 129.0, 129.0 (Ar-C), 127.9 (Ar-C),125.9, 125.9 (C-Ar), 111.9, 109.0 (*C*(CH_3_)_2_), 105.3 (C-1), 104.6 (CI), 82.5 (C-2), 82.1 (C-3), 81.3 (C-4), 77.0 (CH_2_Ar), 72.5 (C-5), 67.4 (C-6), 29.2 (CH_3_Ar), 26.9, 26.8, 26.3, 25.4 (CH_3_); HRMS (ESI-TOF) *m/z* [M + Na]^+^: calcd for C_20_H_27_IO_6_Na: 513.07446, found: 513.07419; Anal calcd for C_20_H_27_IO_6_: C 48.99, H 5.55, found: C 48.92, H 5.79.

**20**: Prepared from **18** (550 mg, 1.18 mmol) and **19** (238 mg, 1.08 mmol) accordingly to general procedure C; colorless oil; 96% (688 mg) yield after column chromatography (PhMe/EtOAc, 40/1 → 10/1); R_f_ = 0.27 (PhMe/EtOAc, 30/1); [α]^20^_D_ = +53.9° (c = 1.0, CH_2_Cl_2_); ^1^H-NMR (400 MHz, CD_2_Cl_2_) δ = 7.77 (dd, *J* = 1.5, 7.7 Hz, 1H, H-Ar), 7.11–7.43 (m, 15H, H-Ar), 6.74 (d, *J* = 7.7 Hz, 2H, H-Ar), 4.99 (d, *J* = 10.9 Hz, 1H, CH_2_Ar), 4.93 (d, *J* = 11.2 Hz, 1H, CH_2_Ar), 4.85 (d, *J* = 10.9 Hz, 1H, CH_2_Ar), 4.74–4.81 (m, 2H, H-1, CH_2_Ar), 4.61–4.72 (m, 2H, CH_2_Ar), 4.18 (dd, *J* = 1.7, 10.3 Hz, 1H, H-6a), 4.10 (dd, *J* = 4.5, 10.3 Hz, 1H, H-6b), 3.91–4.04 (m, 2H, H-4, H-5), 3.78–3.87 (m, 1H, H-3), 3.64 (dd, *J* = 3.5, 9.5 Hz, 1H, H-2), 3.45 (s, 3H, CH_3_); ^13^C-NMR (101 MHz, CD_2_Cl_2_) δ = 157.7, 139.9, 139.5, 139.0, 130.0, 129.5, 128.9, 128.8, 128.7, 128.6, 128.5, 128.4, 128.3, 128.1, 128.1, 123.1, 112.4 (C-Ar), 98.5 (C-1), 86.6 (CI), 82.5 (C-4), 81.0 (C-2), 78.3 (C-3), 76.1, 75.6, 73.6 (CH_2_Ar), 69.8 (C-5), 68.5 (C-6), 55.7 (CH_3_); HRMS (ESI-TOF) *m/z* [M + Na]^+^: calcd for C_34_H_35_IO_6_Na: 689.13705, found: 689.13711; Anal calcd for C_34_H_35_IO_6_: C 61.27, H 5.29, found: C 61.39, H 5.39.

**24**: Prepared from **21** (5.08 g, 26.2 mmol) accordingly to general procedure E; colorless solid; 85% (9.70 g) yield after column chromatography (PhMe/EtOAc, 1/4); R_f_ = 0.30 (PhMe/EtOAc, 1/4); mp = 75 °C (*n*-heptane, EtOAc); [α]^20^_D_ = −44.0° (c = 1.0, CHCl_3_); ^1^H-NMR (400 MHz, CDCl_3_) δ = 7.32–7.43 (m, 5H, H-Ar), 7.04–7.27 (m, 10H, H-Ar), 4.09 (d, *J* = 7.7 Hz, 1H, H-1), 4.03 (bs, 1H, OH), 3.56 (bs, 1H, OH), 3.44 (s, 3H, CH_3_), 3.19–3.41 (m, 7H, OH, H-2, H-3, H-4, H-5, H-6a, H-6b); ^13^C-NMR (101 MHz, CDCl_3_) δ = 143.7, 129.6, 129.0, 128.6, 128.2, 127.9, 127.1, 125.3 (C-Ar), 103.3 (C-1), 86.9 (CPh_3_), 76.3, 74.2, 73.4, 71.6 (C-2, C-3, C-4, C-5), 64.1 (C-6), 56.8 (CH_3_); HRMS (ESI-TOF) *m/z* [M + Na]^+^: calcd for C_26_H_28_O_6_Na: 459.17781, found: 459.17772; Anal calcd for C_26_H_28_O_6_: C 71.54, H 6.47, found: C 71.50, H 6.57.

**25**: Prepared from **22** (5.91 g, 30.0 mmol) accordingly to general procedure E; colorless solid; 71% (9.30 g) yield after column chromatography (PE/EtOAc, 1/2); analytical data were in good accordance with literature values [29].

**26**: Prepared from **23** (5.91 g, 30.0 mmol) accordingly to general procedure E; colorless solid; 65% (8.52 g) yield after column chromatography (PhMe/EtOAc, 1/4 → EtOAc); R_f_ = 0.36 (EtOAc); mp = 127 °C (*n*-heptane, CH_2_Cl_2_); [α]^20^_D_ = +53.0° (c = 1.0, CHCl_3_); ^1^H-NMR (400 MHz, CDCl_3_) δ = 7.30–7.44 (m, 5H, H-Ar), 7.08–7.29 (m, 10H, H-Ar), 4.71 (d, *J* = 3.8 Hz, 1H, H-1), 3.88–3.90 (m, 1H, H-3 or H-4), 3.69–3.76 (m, 2H, H-2, H-5), 3.59–3.66 (m, 1H, H-3 or H-4), 3.31–3.38 (m, 4H, CH_3_, H-6a), 3.28 (dd, *J* = 4.0, 9.6 Hz, 1H, H-6b); ^13^C-NMR (101 MHz, CDCl_3_) δ = 146.8, 143.7, 128.6, 127.9, 127.2, 127.1 (C-Ar), 99.4 (C-1), 87.0 (CPh_3_), 71.1, 69.7, 69.6, 69.1 (C-2, C-3, C-4, C-5), 63.2 (C-6), 55.3 (CH_3_); HRMS (ESI-TOF) *m/z* [M + Na]^+^: calcd for C_26_H_28_O_6_Na: 459.17781, found: 459.17816; Anal calcd for C_26_H_28_O_6_: C 71.54, H 6.47, found: C 71.55, H 6.51.

**27**: Prepared from **24** (3.64 g, 8.34 mmol) and BnBr (6.42 g, 37.5 mmol) accordingly to general procedure A; colorless syrup; 76% (4.50 g) yield after column chromatography (PE/EtOAc, 14/1 → PE/EtOAc, 3/1); R_f_ = 0.27 (PE/EtOAc, 14/1); [α]^20^_D_ = +3.9° (c = 1.0, CHCl_3_); ^1^H-NMR (400 MHz, CDCl_3_) δ = 7.38–7.52 (m, 6H, H-Ar), 7.05–7.35 (m, 22H, H-Ar), 6.73–6.83 (m, 2H, H-Ar), 4.90 (d, *J* = 11.0 Hz, 1H, CH_2_Ph), 4.82 (d, *J* = 10.6 Hz, 1H, CH_2_Ph), 4.66–4.76 (m, 2H, CH_2_Ph), 4.62 (d, *J* = 10.3 Hz, 1H, CH_2_Ph), 4.24–4.36 (m, 2H, CH_2_Ph, H-1), 3.71–3.83 (m, 1H, H-4), 3.59 (s, 3H, CH_3_), 3.43–3.57 (m, 3H, H-2, H-3, H-6a), 3.30–3.36 (m, 1H, H-6), 3.17 (dd, *J* = 3.8, 10.1 Hz, 1H, H-6b); ^13^C-NMR (101 MHz, CDCl_3_) δ = 144.0, 138.7, 138.6, 137.9, 128.9, 128.5, 128.4, 128.3, 128.2, 128.1, 127.8, 127.8, 127.7 (C-Ar), 104.6 (C-1), 86.4 (CPh_3_), 84.7 (C-3), 82.7 (C-2), 77.9 (C-4), 76.0, 75.1, 74.9 (CH_2_Ph), 74.6 (C-5), 62.4 (C-6), 56.7 (CH_3_); HRMS (ESI-TOF) *m/z* [M + Na]^+^: calcd for C_47_H_46_O_6_Na: 729.31866, found: 729.31931; Anal calcd for C_47_H_46_O_6_: C 79.86, H 6.56, found: C 79.84, H 6.66.

**28**: Prepared from **25** (3.86 g, 8.84 mmol) and BnBr (6.80 g, 39.8 mmol) accordingly to general procedure A; colorless solid; 81% (5.04 g) yield after column chromatography (PE/EtOAc, 9/1); R_f_ = 0.52 (PE/EtOAc, 4/1); mp = 112 °C (*n*-heptane, CH_2_Cl_2_); [α]^20^_D_ = +17.8° (c = 1.0, CHCl_3_); ^1^H-NMR (400 MHz, CDCl_3_) δ = 7.40–7.49 (m, 6H, H-Ar), 7.36 (dd, *J* = 1.5, 7.8 Hz, 2H, H-Ar), 7.05–7.30 (m, 20H, H-Ar), 6.81 (dd, *J* = 1.5, 7.8 Hz, 2H, H-Ar), 4.7–4.80 (m, 2H, CH_2_Ph, H-1), 4.61–4.69 (m, 2H, CH_2_Ph), 4.56 (s, 2H, CH_2_Ph), 4.19 (d, *J* = 10.5 Hz, 1H, CH_2_Ph), 3.90–3.97 (m, 1H, H-4), 3.80 (dd, *J* = 3.2, 9.3 Hz, 1H, H-3), 3.74 (dd, *J* = 1.9, 3.2 Hz, 1H, H-2), 3.66–3.72 (m, 1H, H-5), 3.44 (dd, *J* = 1.7, 9.8 Hz, 1H, H-6a), 3.30 (s, 3H, CH_3_), 3.19 (dd, *J* = 5.3, 9.8 Hz, 1H, H-6b); ^13^C-NMR (101 MHz, CDCl_3_) δ = 144.2, 138.6, 138.6, 138.2, 128.8, 128.3, 128.3, 128.1, 127.7, 127.6, 127.5, 127.4, 127.4, 126.8 (C-Ar), 98.7 (C-1), 86.2 (CPh_3_), 80.2 (C-3), 75.4 (C-2), 75.0 (C-4), 72.7, 72.2, 71.7 (CH_2_Ph), 63.0 (C-6), 54.5 (CH_3_); HRMS (ESI-TOF) *m/z* [M + Na]^+^: calcd for C_47_H_46_O_6_Na: 729.31866, found: 729.31900; Anal calcd for C_47_H_46_O_6_: C 79.86, H 6.56, found: C 79.75, H 6.62.

**29**: Prepared from **26** (3.69 g, 8.45 mmol) and BnBr (6.50 g, 38.0 mmol) accordingly to general procedure A; colorless syrup; 81% (4.81 g) yield after column chromatography (PE/EtOAc, 9/1 → 3/1); R_f_ = 0.36 (PE/EtOAc, 6/1); [α]^20^_D_ = +20.2° (c = 1.0, CHCl_3_); ^1^H-NMR (400 MHz, CDCl_3_) δ = 7.25–7.37 (m, 12H, H-Ar), 7.07–7.24 (m, 16H, H-Ar), 6.97–7.05 (m, 2H, H-Ar), 4.69–4.82 (m, 3H, CH_2_Ph), 4.52–4.67 (m, 3H, CH_2_Ph, H-1), 4.40 (d, *J* = 11.4 Hz, 1H, CH_2_Ph), 3.89 (dd, *J* = 3.7, 9.9, 1H, H-2), 3.77–3.84 (m, 2H, H-3, H-4), 3.63 (m, 1H, H-5), 3.33 (dd, *J* = 6.2, 9.7 Hz, 1H, H-6a), 3.29 (s, 3H, CH_3_), 3.05–3.09 (m, 1H, H-6b); ^13^C-NMR (101 MHz, CDCl_3_) δ = 143.9, 138.9, 138.5, 138.5, 128.6, 128.3, 128.3, 128.1, 128.1, 128.0, 127.8, 127.6, 127.5, 127.5, 127.4, 127.0 (C-Ar), 98.6 (C-1), 86.9 (CPh_3_), 79.0 (C-3), 76.4 (C-2), 75.6 (C-4), 74.6, 73.5, 73.3 (CH_2_Ph), 69.5 (C-5), 63.1 (C-6), 55.1 (CH_3_); HRMS (ESI-TOF) *m/z* [M + Na]^+^: calcd for C_47_H_46_O_6_Na: 729.31866, found: 729.31864; Anal calcd for C_47_H_46_O_6_: C 79.86, H 6.56, found: C 79.87, H 6.57.

**30**: Prepared from **27** (4.30 g, 6.08 mmol) accordingly to general procedure F; colorless solid; 56% (1.58 g) yield after column chromatography (PE/EtOAc, 3/1 → 2/1); analytical data were in good accordance with literature values [30].

**31**: Prepared from **28** (3.14 g, 4.44 mmol) accordingly to general procedure F; colorless solid; 76% (1.56 g) yield after column chromatography (PE/EtOAc, 3/1 → 2/1); analytical data were in good accordance with literature values [31]. 

**32**: Prepared from **29** (4.31 g, 6.10 mmol) accordingly to general procedure F; colorless syrup; 71% (2.00 g) yield after column chromatography (PE/EtOAc, 2/1 → 1/2); analytical data were in good accordance with literature values [31].

**34**: Prepared from **30** (585 mg, 1.26 mmol) and **33** (135 mg, 0.572 mmol) accordingly to general procedure C; colorless solid; 74% (480 mg) yield after column chromatography (PhMe/EtOAc, 10/1 → 6/1); R_f_ = 0.55 (PhMe/EtOAc, 6/1); mp = 145 °C (*n*-heptane, CH_2_Cl_2_); [α]^20^_D_ = +49.4° (c = 1.0, CH_2_Cl_2_); ^1^H-NMR (400 MHz, CD_2_Cl_2_) δ = 7.15–7.43 (m, 31H, H-Ar), 6.49 (d, *J* = 8.3 Hz, 2H, H-Ar), 4.88–4.99 (m, 6H, CH_2_Ph), 4.83 (d, *J* = 11.1 Hz, 2H, CH_2_Ph), 4.74 (d, *J* = 11.1 Hz, 2H, CH_2_Ph), 4.65 (d, *J* = 11.1 Hz, 2H, CH_2_Ph), 4.39 (d, *J* = 7.8 Hz, 2H, H-1), 4.32 (dd, *J* = 1.6, 10.4 Hz, 2H, H-6a), 4.17 (dd, *J* = 4.3, 10.4 Hz, 2H, H-6b), 3.89–3.99 (m, 2H, H-4), 3.67–3.73 (m, 2H, H-3), 3.60–3.66 (m, 2H, H-5), 3.57 (s, 6H, CH_3_), 3.48 (dd, *J* = 7.8, 9.0 Hz, 2H, H-2); ^13^C-NMR (101 MHz, CH_2_Cl_2_) δ = 159.2, 139.4, 139.4, 139.0, 130.3, 128.8, 128.6, 128.5, 128.5, 128.1, 128.1, 105.9 (C-Ar), 105.4 (C-1), 85.1 (C-3), 82.8 (C-2), 79.0 (CI), 78.2 (C-4), 76.0, 75.6, 75.0 (CH_2_ Ph), 74.2 (C-5), 68.6 (C-6), 57.5 (CH_3_); HRMS (ESI-TOF) *m/z* [M + Na]^+^: calcd for C_62_H_65_IO_12_Na: 1151.34130, found: 1151.34152; Anal calcd for C_62_H_65_IO_12_: C 65.95, H 5.80, found: C 65.92, H 5.83.

**35**: Prepared from **31** (470 mg, 1.01 mmol) and **33** (109 mg, 0.460 mmol) accordingly to general procedure C; slight yellow syrup; 29% (153 mg) yield after column chromatography (PhMe/EtOAc, 15/1 → 6/1); R_f_ = 0.55 (PhMe/EtOAc, 6/1); [α]^20^_D_ = +31.5° (c = 1.0, CH_2_Cl_2_); ^1^H-NMR (400 MHz, CD_2_Cl_2_) δ = 7.14–7.48 (m, 31H, H-Ar), 6.43 (d, *J* = 8.3 Hz, 2H, H-Ar), 4.96 (d, *J* = 11.1 Hz, 2H, CH_2_Ph), 4.83 (d, *J* = 1.6 Hz, 2H, H-1), 4.65–4.80 (m, 8H, CH_2_Ph), 4.63 (d, *J* = 11.2 Hz, 2H, CH_2_Ph), 4.24 (dd, *J* = 1.5, 10.2 Hz, 2H, H-6a), 4.09–4.21 (m, 4H, H-5, H-6b), 3.85–3.96 (m, 6H, H-2, H-3, H-4), 3.40 (s, 6H, CH_3_); ^13^C-NMR (101 MHz, CH_2_Cl_2_) δ = 159.3, 139.3, 139.1, 130.2, 128.9, 128.8, 128.8, 128.6, 128.4, 128.2, 128.1, 128.1, 128.0, 105.4 (C-Ar), 99.6 (C-1), 80.9 (C-3 or C-4), 78.7 (CI), 75.8 (C-2), 75.6 (CH_2_Ph), 75.4 (C-5), 73.4, 72.5 (CH_2_Ph), 71.2 (C-3 or C-4), 69.1 (C-6), 55.4 (CH_3_); HRMS (ESI-TOF) *m/z* [M + Na]^+^: calcd for C_62_H_65_IO_12_Na: 1151.34130, found: 1151.34185; Anal calcd for C_62_H_65_IO_12_: C 65.95, H 5.80, found: C 66.00, H 5.85.

**36**: Prepared from **32** (568 mg, 1.22 mmol) and **33** (116 mg, 0.490 mmol) accordingly to general procedure D; slight yellow syrup; 57% (315 mg) yield after column chromatography (PhMe/EtOAc, 15/1 → 10/1); R_f_ = 0.58 (PhMe/EtOAc, 6/1); [α]^20^_D_ = +19.8° (c = 1.0, CH_2_Cl_2_); ^1^H-NMR (400 MHz, CD_2_Cl_2_) δ = 7.42–7.48 (m, 4H, H-Ar), 7.11–7.41 (m, 28H, H-Ar), 6.41 (d, *J* = 8.3 Hz, 2H, H-Ar), 4.99 (d, *J* = 11.0 Hz, 2H, CH_2_Ph), 4.89 (d, *J* = 12.0 Hz, 2H, CH_2_Ph), 4.78–4.84 (m, 6H, CH_2_Ph, H-1), 4.65 (d, *J* = 11.7 Hz, 2H, CH_2_Ph), 4.54 (d, *J* = 11.0 Hz, 2H, CH_2_Ph), 4.21–4.24 (m, 2H, H-3), 4.14–4.20 (m, 2H, H-5), 3.89–4.12 (m, 8H, H-2, H-4, H-6a, H-6b), 3.43 (s, 6H, CH_3_); ^13^C-NMR (101 MHz, CH_2_Cl_2_) δ = 158.9, 139.5, 139.4, 139.2, 130.5, 129.5, 128.9, 128.9, 128.8, 128.8, 128.7, 128.4, 128.2, 128.1, 128.1, 105.5 (C-Ar), 99.3 (C-1), 79.1 (C-2 or C-4), 78.2 (CI), 77.2 (C-2 or C-4), 76.0 (C-3), 75.6, 73.8, 73.6 (CH_2_Ph), 69.0 (C-5), 68.3 (C-6), 55.9 (CH_3_); HRMS (ESI-TOF) *m/z* [M + Na]^+^: calcd for C_62_H_65_IO_12_Na: 1151.34130, found: 1151.34058, Anal calcd for C_62_H_65_IO_12_: C 65.95, H 5.80; found: C 65.85, H 6.04.

**38**: Prepared from **37** (2.02 g, 4.35 mmol) and 3,5-dimethyl-benzyl bromide (1.30 g, 6.53 mmol) accordingly to general procedure A; colorless solid; 97% (2.47 g) yield after column chromatography (PE/EtOAc, 5/1 → 4/1); R_f_ = 0.29 (PE/EtOAc, 5/1); mp = 53 °C (*n*-heptane, CH_2_Cl_2_); [α]^20^_D_ = +81.7° (c = 1.0, CHCl_3_); ^1^H-NMR (400 MHz, CDCl_3_) δ = 7.37–7.48 (m, 6H, H-Ar), 7.09–7.27 (m, 9H, H-Ar), 6.79 (s, 1H, H-Ar), 6.50 (s, 2H, H-Ar), 4.87 (d, *J* = 3.6 Hz, 1H, H-1), 4.50 (d, *J* = 10.0 Hz, 1H, CH_2_Ar), 4.15 (d, *J* = 10.0 Hz, 1H, CH_2_Ar), 3.63 – 3.71 (m, 1H, H-5), 3.59 (s, 3H, CH_3_O), 3.48–3.56 (m, 5H, CH_3_O, H-3, H-4), 3.43 (dd, *J* = 1.8, 10.0 Hz, 1H, H-6a), 3.37 (s, 3H, CH_3_O), 3.27 (dd, *J* = 3.6, 9.3 Hz, 1H, H-2), 3.13 (dd, *J* = 4.3, 10.0 Hz, 1H, H-6b), 2.16 (s, 6H, CH_3_Ar); ^13^C-NMR (101 MHz, CHCl_3_) δ = 144.0, 137.7, 137.7, 129.3, 128.8, 127.8, 126.9, 126.0 (C-Ar), 97.3 (C-1), 86.2 (CPh_3_), 83.7 (C-3), 82.0 (C-2), 77.9 (C-4), 75.0 (CH_2_Ar), 70.1 (C-5), 62.4 (C-6), 61.2, 59.0, 54.8 (CH_3_O), 21.2 (CH_3_Ar); HRMS (ESI-TOF) *m/z* [M + Na]^+^: calcd for C_37_H_42_O_6_Na: 605.28736, found: 605.28801; Anal calcd for C_37_H_42_O_6_: C 76.26, H 7.27, found: C 76.05, H 7.52.

**39**: Prepared from **37** (1.82 g, 3.91 mmol) and 3,5-di*^t^*butyl-4-methoxy-benzyl bromide (1.47 g, 4.69 mmol) accordingly to general procedure A; colorless solid; 84% (2.29 g) yield after column chromatography (PE/EtOAc, 5/1 → 4/1); R_f_ = 0.37 (PE/EtOAc, 5/1); mp = 69 °C (*n*-heptane, CH_2_Cl_2_); [α]^20^_D_ = +49.3° (c = 1.0, CHCl_3_); ^1^H-NMR (400 MHz, CDCl_3_) δ = 7.39–7.48 (m, 6H, H-Ar), 7.10–7.27 (m, 9H, H-Ar), 6.88 (s, 2H, H-Ar), 4.88 (d, *J* = 3.5 Hz, 1H, H-1), 4.45 (d, *J* = 9.7 Hz, 1H, CH_2_Ar), 4.05 (d, *J* = 9.7 Hz, 1H, CH_2_Ar), 3.63–3.70 (m, 1H, H-5), 3.62 (s, 3H, CH_3_O), 3.49–3.60 (m, 8H, 2 × CH_3_O, H-3, H-4), 3.46 (dd, *J* = 1.7, 10.1 Hz, 1H, H-6a), 3.36 (s, 3H, CH_3_O), 3.29 (dd, *J* = 3.5, 9.2 Hz, 1H, H-2), 3.14 (dd, *J* = 3.9, 10.1 Hz, 1H, H-6b), 1.29 (s, 18H, C(CH_3_)_3_); ^13^C-NMR (101 MHz, CHCl_3_) δ = 159.1, 144.0, 143.3, 132.0, 128.8, 127.7, 127.0, 126.8 (C-Ar), 97.3 (C-1), 86.2 (CPh_3_), 83.5 (C-3), 82.3 (C-2), 78.1 (C-4), 75.7 (CH_2_Ar), 70.1 (C-5), 64.2 (CH_3_O), 62.4 (C-6), 61.4, 59.0, 54.9 (CH_3_O), 35.6 (*C*(CH_3_)_3_), 32.0 (C(*C*H_3_)_3_); HRMS (ESI-TOF) *m/z* [M + Na]^+^: calcd for C_44_H_56_O_7_Na: 719.39182, found: 719.39276; Anal calcd for C_44_H_56_O_7_: C 75.83, H 8.10, found: C 75.83, H 8.36.

**40**: Prepared from **37** (2.02 g, 4.35 mmol) and 1-(bromomethyl)-naphthalene (1.44 g, 6.53 mmol) accordingly to general procedure A; colorless solid; 99% (2.60 g) yield after column chromatography (PE/EtOAc, 5/1 → 4/1); R_f_ = 0.19 (PE/EtOAc, 5/1); mp = 65 °C (*n*-heptane, CH_2_Cl_2_); [α]^20^_D_ = +95.5° (c = 1.0, CHCl_3_); ^1^H-NMR (400 MHz, CDCl_3_) δ = 7.78 (d, *J* = 8.3 Hz, 1H, H-Ar), 7.74 (d, *J* = 8.1 Hz, 1H, H-Ar), 7.67 (d, *J* = 8.2 Hz, 1H, H-Ar), 7.30–7.43 (m, 7H, H-Ar), 7.07–7.28 (m, 11H, H-Ar), 6.95 (d, *J* = 6.5 Hz, 1H, H-Ar), 5.10 (d, *J* = 11.0 Hz, 1H, CH_2_Ar), 4.87 (d, *J* = 3.6 Hz, 1H, H-1), 4.63 (d, *J* = 11.1 Hz, 1H, CH_2_Ar), 3.67–3.75 (m, 1H, H-5), 3.58–3.63 (m, 1H, H-4), 3.48–3.57 (m, 7H, 2 × CH_3_O, H-3), 3.44 (dd, *J* = 1.8, 10.1 Hz, 1H, H-6a), 3.38 (s, 3H, CH_3_O), 3.30 (dd, *J* = 3.6, 9.4 Hz, 1H, H-2), 3.08 (dd, *J* = 4.7, 10.1 Hz, 1H, H-6b); ^13^C-NMR (101 MHz, CHCl_3_) δ = 143.9, 133.7, 133.5, 131.4, 128.7, 128.3, 128.3, 127.7, 126.9, 126.6, 126.1, 125.5, 125.1, 123.9 (C-Ar), 97.1 (C-1), 86.3 (CPh_3_), 83.5 (C-3), 82.5 (C-2), 77.8 (C-4), 72.6 (CH_2_Ar), 70.2 (C-5), 62.7 (C-6), 61.3, 58.9, 54.9 (CH_3_O); HRMS (ESI-TOF) *m/z* [M + Na]^+^: calcd for C_39_H_40_O_6_Na: 627.27171, found: 627.27231; Anal calcd for C_39_H_40_O_6_: C 77.46, H 6.67, found: C 77.51, H 7.01.

**41**: Prepared from **38** (2.34 g, 4.02 mmol) accordingly to general procedure F; colorless solid; 78% (1.07 g) yield after column chromatography (PE/EtOAc, 1/1 → 1/2); R_f_ = 0.25 (PE/EtOAc, 1/1); mp = 109 °C (*n*-heptane, CH_2_Cl_2_); [α]^20^_D_ = +115.6° (c = 1.0, CHCl_3_); ^1^H-NMR (400 MHz, CDCl_3_) δ = 6.89–7.02 (m, 3H, H-Ar), 4.78–4.86 (m, 2H, CH_2_Ar, H-1), 4.58 (d, *J* = 10.8 Hz, 1H, CH_2_Ar), 3.80 (dd, *J =* 2.8, 11.7 Hz, 1H, H-6a), 3.73 (dd, *J =* 3.9, 11.7 Hz 1H), 3.59–3.69 (m, 5H, CH_3_O, H-3, H-4), 3.54 (s, 3H, CH_3_O), 3.36–3.47 (m, 4H, CH_3_O, H-4), 3.22 (dd, *J* = 3.5, 9.5 Hz, 1H, H-2), 2.32 (s, 6H, CH_3_Ar); ^13^C-NMR (101 MHz, CHCl_3_) δ = 138.1, 137.9, 129.5, 126.0 (C-Ar), 97.5 (C-1), 83.6 (C-3), 82.0 (C-2), 77.2 (C-4), 75.0 CH_2_Ar, 70.6 (C-5), 61.9 (C-6), 61.0, 59.0, 55.1 (CH_3_O), 21.2 (CH_3_Ar); HRMS (ESI-TOF) *m/z* [M + Na]^+^: calcd for C_18_H_28_O_6_Na: 363.17781, found: 363.17822; Anal calcd for C_18_H_28_O_6_: C 63.51, H 8.29, found: C 63.46, H 8.40.

**42**: Prepared from **39** (2.15 g, 3.09 mmol) accordingly to general procedure F; colorless solid; 78% (1.10 g) yield after column chromatography (PE/EtOAc, 1/1 → 1/2); R_f_ = 0.20 (PE/EtOAc, 1/1); mp = 139 °C (*n*-heptane, CH_2_Cl_2_); [α]^20^_D_ = +92.7° (c = 1.0, CHCl_3_); ^1^H-NMR (400 MHz, CDCl_3_) δ = 7.24 (s, 2H, H-Ar), 4.77–4.85 (m, 2H, CH_2_Ar, H-1), 4.56 (d, *J* = 10.5 Hz, 1H, CH_2_Ar), 3.78 (dd, *J* = 2.8, 11.8 Hz, 1H, H-6a) 3.60–3.74 (m, 9H, 2 × CH_3_O, H-3, H-5, H-6b), 3.55 (s, 3H, CH_3_O), 3.40–3.45 (m, 4H, CH_3_O, H-4), 3.23 (dd, *J* = 3.6, 9.5 Hz, 1H, H-2), 1.43 (s, 18H, C(CH_3_)_3_); ^13^C-NMR (101 MHz, CHCl_3_) δ = 159.4, 143.8, 131.9, 126.9 (C-Ar), 97.5 (C-1), 83.7 (C-3), 82.1 (C-2), 77.2 (C-4), 75.5 (CH_2_Ar), 70.5 (C-5), 64.2 (CH_3_O), 61.8 (C-6), 61.1, 59.0, 55.1 (CH_3_O), 35.7 (*C*(CH_3_)_3_), 32.0 (C(*C*H_3_)_3_); HRMS (ESI-TOF) *m/z* [M + Na]^+^: calcd for C_25_H_42_O_7_Na: 477.28277, found: 477.28230; Anal calcd for C_25_H_42_O_7_: C 66.05, H 9.31, found: C 66.06, H 9.53.

**43**: Prepared from **40** (2.46 g, 4.7 mmol) accordingly to general procedure F; colorless solid; 80% (1.18 g) yield after column chromatography (PE/EtOAc, 1/1 → 1/2); R_f_ = 0.19 (PE/EtOAc, 1/1); mp = 74 °C (*n*-heptane, CH_2_Cl_2_); [α]^20^_D_ = +138.9° (c = 1.0, CHCl_3_); ^1^H-NMR (400 MHz, CDCl_3_) δ = 8.16 (d, *J* = 8.4 Hz, 1H, H-Ar), 7.79–7.92 (m, 2H, H-Ar), 7.41–7.60 (m, 4H, H-Ar), 5.41 (d, *J* = 11.4 Hz, 1H, CH_2_Ar), 5.08 (d, *J* = 11.4 Hz, 1H, CH_2_Ar), 4.83 (d, *J* = 3.6 Hz, 1H, H-1), 3.51–3.73 (m, 11H, 2 × CH_3_O, H-3, H-4, H-5, H-6a, H-6b), 3.39 (s, 3H, CH_3_O), 3.27 (dd, *J* = 3.6, 9.5 Hz, 1H, H-2); ^13^C-NMR (101 MHz, CHCl_3_) δ = 133.8, 133.7, 131.6, 128.8, 128.6, 126.9, 126.3, 125.8, 125.3, 123.9 (C-Ar), 97.4 (C-1), 83.6 (C-3), 82.3 (C-2), 76.8 (C-4), 72.7 (CH_2_Ar), 70.5 (C-5), 61.7 (C-6), 61.1, 58.9, 55.1 (CH_3_O); HRMS (ESI-TOF) *m/z* [M + Na]^+^: calcd for C_20_H_26_O_6_Na: 385.16216, found: 385.16198; Anal calcd for C_20_H_26_O_6_: C 66.28, H 7.23, found: C 66.23, H 7.36.

**44**: Prepared from **41** (561 mg, 1.65 mmol) and **33** (194 mg, 0.824 mmol) accordingly to general procedure C; colorless solid; 86% (624 mg) yield after column chromatography (PE/EtOAc, 4/1 → 1/1); R_f_ = 0.26 (PE/EtOAc, 1/1); mp = 107 °C (*n*-heptane, CH_2_Cl_2_); [α]^20^_D_ = +120.0° (c = 1.0, CH_2_Cl_2_); ^1^H-NMR (400 MHz, CD_2_Cl_2_) δ = 7.22 (t, *J* = 8.3 Hz, 1H, H-Ar), 6.94 (s, 2H, H-Ar), 6.78 (s, 4H, H-Ar), 6.41 (d, *J* = 8.3 Hz, 2H, H-Ar), 4.86 (d, *J* = 3.6 Hz, 2H, H-1), 4.80 (d, *J* = 11.0 Hz, 2H, CH_2_Ar), 4.51 (d, *J* = 11.0 Hz, 2H, CH_2_Ar), 4.17 (dd, *J* = 1.6, 10.3 Hz, 2H, H-6a), 4.07 (dd, *J* = 4.4, 10.3 Hz, 2H, H-6b), 3.81–3.88 (m, 2H, H-5), 3.68–3.77 (m, 2H, H-6), 3.63 (s, 6H, CH_3_O), 3.52–3.58 (m, 2H, H-3), 3.48 (s, 6H, CH_3_O), 3.43 (s, 6H, CH_3_O), 3.29 (dd, *J* = 3.6, 9.5 Hz, 2H, H-2), 2.21 (s, 12H, CH_3_Ar); ^13^C-NMR (101 MHz, CH_2_Cl_2_) δ = 159.2, 138.9, 138.4, 130.3, 129.7, 126.6, 105.4 (C-Ar), 98.1 (C-1), 84.3 (C-3), 82.5 (C-2), 78.5 (CI), 78.1 (C-4), 75.6 (CH_2_Ar), 69.8 (C-5), 68.7 (C-6), 61.2, 58.9, 55.6 (CH_3_O), 21.5 (CH_3_Ar); HRMS (ESI-TOF) *m/z* [M + Na]^+^: calcd for C_42_H_57_IO_12_Na: 903.27870, found: 903.27835; Anal calcd for C_42_H_57_IO_12_: C 57.27, H 6.52, found: C 57.05, H 6.55.

**45**: Prepared from **42** (636 mg, 1.40 mmol) and **33** (157 mg, 0.661 mmol) accordingly to general procedure C; colorless solid; 89% (660 mg) yield after column chromatography (PE/EtOAc, 3/1 → 2/1); R_f_ = 0.55 (PE/EtOAc, 1/1); mp = 69 °C (*n*-heptane, CH_2_Cl_2_); [α]^20^_D_ = +87.6° (c = 1.0, CH_2_Cl_2_); ^1^H-NMR (400 MHz, CD_2_Cl_2_) δ = 7.21 (t, *J* = 8.2 Hz, 1H, H-Ar), 7.11 (s, 4H, H-Ar), 6.47 (d, *J* = 8.2 Hz, 2H, H-Ar), 4.86 (d, *J* = 3.6 Hz, 2H, H-1), 4.78 (d, *J* = 10.4 Hz, 2H, CH_2_Ar), 4.48 (d, *J* = 10.4 Hz, 2H, CH_2_Ar), 4.22 (dd, *J* = 1.6, 10.3 Hz, 2H, H-6a), 4.16 (dd, *J* = 4.5, 10.3 Hz, 2H, H-6b), 3.82–3.89 (m, 2H, H-5), 3.70–3.79 (m, 2H, H-4), 3.65 (s, 6H, CH_3_O), 3.63 (s, 6H, CH_3_O), 3.54–3.59 (m, 2H, H-3), 3.48 (s, 6H, CH_3_O), 3.42 (s, 6H, CH_3_O), 3.30 (dd, *J* = 3.6, 9.5 Hz, 2H, H-2), 1.36 (s, 36H, C(CH_3_)_3_); ^13^C-NMR (101 MHz, CH_2_Cl_2_) δ = 159.7, 159.2, 144.1, 133.0, 130.5, 127.3, 105.6 (C-Ar), 98.1 (C-1), 84.3 (C-3), 82.5 (C-2), 78.6 (CI), 78.2 (C-4), 76.1 (CH_2_Ar), 69.8 (C-5), 68.8 (C-6), 64.8, 61.2, 58.9, 55.6 (CH_3_O), 36.1 (*C*(CH_3_)_3_), 32.4 (C(*C*H_3_)_3_); HRMS (ESI-TOF) *m/z* [M + Na]^+^: calcd for C_56_H_85_IO_14_Na: 1131.48763, found: 1131.48813; Anal calcd for C_56_H_85_IO_14_: C 60.64, H 7.72, found: C 60.59, H 8.02.

**46**: Prepared from **43** (600 mg, 1.66 mmol) and **33** (178 mg, 0.750 mmol) accordingly to general procedure C; colorless solid; 78% (541 mg) yield after column chromatography (PE/EtOAc, 2/1 → 1/1); R_f_ = 0.26 (PE/EtOAc, 1/1); mp = 78 °C (*n*-heptane, CH_2_Cl_2_); [α]^20^_D_ = +120.3° (c = 1.0, CH_2_Cl_2_); ^1^H-NMR (400 MHz, CD_2_Cl_2_) δ = 8.02 (d, *J* = 8.4 Hz, 2H, H-Ar), 7.83 (d, *J* = 8.2 Hz, 2H, H-Ar), 7.75 (d, *J* = 7.9 Hz, 2H, H-Ar), 7.26–7.48 (m, 8H, H-Ar), 7.08 (t, *J* = 8.3 Hz, 1H, H-Ar), 6.08 (d, *J* = 8.3 Hz, 2H, H-Ar), 5.39 (d, *J* = 11.4 Hz, 2H, CH_2_Ar), 5.05 (d, *J* = 11.4 Hz, 2H, CH_2_Ar), 4.86 (d, *J* = 3.5 Hz, 2H, H-1), 4.04 (dd, *J* = 1.2, 10.0 Hz, 2H, H-6a), 3.85–3.94 (m, 4H, H-4, H-6a), 3.80–3.85 (m, 2H, H-5), 3.68 (s, 6H, CH_3_), 3.60–3.64 (m, 2H, H-3), 3.49 (s, 6H, CH_3_), 3.41 (s, 6H, CH_3_), 3.34 (dd, *J* = 3.5, 9.5 Hz, 2H, H-2); ^13^C-NMR (101 MHz, CH_2_Cl_2_) δ = 158.9, 134.6, 134.2, 132.3, 130.3, 129.1, 129.0, 127.8, 126.7, 126.2, 125.8, 124.7, 105.3 (C-Ar), 98.0 (C-1), 84.4 (C-3), 82.8 (C-2), 78.4 (CI), 77.4 (C-4), 73.3 (CH_2_Ar), 69.6 (C-5), 68.5 (C-6), 61.2, 58.8, 55.6 (CH_3_); HRMS (ESI-TOF) *m/z* [M + Na]^+^: calcd for C_46_H_53_IO_12_Na: 947.24740, found: 947.24650; Anal calcd for C_46_H_53_IO_12_: C 59.74, H 5.78, found: C 59.81, H 6.08.

**48**: Prepared from **47** (3.00 g, 6.87 mmol) and 1-(bromomethyl)-naphthalene (5.32 g, 24.1 mmol) accordingly to general procedure A; colorless solid; 79% (4.65 g) yield after column chromatography (PhMe → PhMe/EtOAc, 20/1); R_f_ = 0.26 (PhMe); mp = 71 °C (PhMe, EtOH); [α]^20^_D_ = +58.2° (c = 1.0, PhMe); ^1^H-NMR (400 MHz, PhMe-d_8_) δ = 7.87 (d, *J* = 8.3 Hz, 1H, H-Ar), 7.46–7.62 (m, 13H, H-Ar), 7.39 (dd, *J* = 6.7, 17.2 Hz, 2H, H-Ar), 6.87–7.28 (m, 28H, H-Ar), 5.42 (d, *J* = 11.6 Hz, 1H, CH_2_Ar), 5.25 (d, *J* = 12.1 Hz, 1H, CH_2_Ar), 5.05 (d, *J* = 11.8 Hz, 1H, CH_2_Ar), 4.98 (d, *J* = 11.8 Hz, 1H, CH_2_Ar), 4.87 (d, *J* = 11.8 Hz, 1H, CH_2_Ar), 4.79 (d, *J* = 12.1 Hz, 1H, CH_2_Ar), 4.73 (d, *J* = 3.5 Hz, 1H, H-1), 4.31 (t, *J* = 9.2 Hz, 1H, H-3), 4.01–4.06 (m, 1H, H-5), 3.83–3.91 (m, 1H, H-4), 3.80 (dd, *J* = 3.5, 9.5 Hz, 1H, H-2), 3.61 (dd, *J* = 1.7, 9.9 Hz, 1H, H-6a), 3.31 (dd, *J* = 5.0, 9.9 Hz, 1H, H-6b), 3.23 (s, 3H, CH_3_); ^13^C-NMR (101 MHz, PhMe-d_8_) δ = 144.7, 135.5, 134.8, 134.7, 134.2, 134.0, 133.8, 132.3, 132.0, 131.5, 129.3, 129.2, 129.1, 128.6, 128.5, 128.2, 128.0, 127.7, 127.1, 126.5, 126.2, 125.9, 125.9, 125.8, 125.8, 125.6, 125.5, 125.5, 125.4, 125.3, 124.9, 124.5, 123.9 (C-Ar), 98.3 (C-1), 86.9 (CPh_3_), 82.3 (C-3), 81.6 (C-2), 78.8 (C-4), 73.7, 72.6, 71.5 (CH_2_Ar), 71.0 (C-5), 63.6 (C-6), 54.8 (CH_3_); HRMS (ESI-TOF) *m/z* [M + Na]^+^: calcd for C_59_H_52_O_6_Na: 879.36561, found: 879.36644; Anal calcd for C_59_H_52_O_6_: C 82.68, H 6.12, found: C 82.45, H 6.34.

**49**: Prepared from **48** (4.45 g, 5.19 mmol) accordingly to general procedure F; colorless solid; 69% (2.19 g) yield after column chromatography (PhMe/EtOAc, 5/1); R_f_ = 0.31 (PhMe/EtOAc, 5/1); mp = 67 °C (CH_2_Cl_2_, EtOH); [α]^20^_D_ = +23.0° (c = 1.0, CHCl_3_); ^1^H-NMR (400 MHz, CDCl_3_) δ = 8.12 (dd, *J* = 1.0 8.0 Hz, 1H, H-Ar), 7.96 (d, *J* = 8.4 Hz, 1H, H-Ar), 7.80 (d, *J* = 8.3 Hz, 1H, H-Ar), 7.62–7.76 (m, 6H, H-Ar), 7.11–7.43 (m, 13H, H-Ar), 5.44 (d, *J* = 11.5 Hz, 1H, CH_2_Ar), 5.12–5.29 (m, 3H, CH_2_Ar), 4.90–4.99 (m, 2H, CH_2_Ar), 4.23 (d, *J* = 3.5 Hz, 1H, H-1), 4.09–4.15 (m, 1H, H-3), 3.55–3.66 (m, 3H, H-2, H-4, H-5), 3.46 – 3.53 (m, 1H, H-6a), 3.41 (dd, *J* = 3.4, 10.9 Hz, 1H, H-6b), 3.07 (s, 3H, CH_3_); ^13^C-NMR (101 MHz, CHCl_3_) δ = 134.4, 133.8, 133.7, 133.5, 133.5, 133.4, 131.7, 131.3, 131.2, 129.1, 128.5, 128.2, 127.2, 126.2, 126.2, 126.1, 126.0, 125.8, 125.7, 125.7, 125.5, 125.3, 125.2, 125.1, 124.3, 123.8, 123.6 (C-Ar), 98.1 (C-1), 82.0 (C-2), 79.7 (C-3), 77.1 (C-4), 73.5, 72.6, 72.0 (CH_2_Ar), 70.6 (C-5), 61.7 (C-6), 55.1 (CH_3_); HRMS (ESI-TOF) *m/z* [M + Na]^+^: calcd for C_40_H_38_O_6_Na: 637.2560677, found: 637.25562; Anal calcd for C_40_H_38_O_6_: C 78.15, H 6.23, found: C 78.13, H 6.28.

**50**: Prepared from **49** (1062 mg, 1.73 mmol) and **33** (186 mg, 0.786 mmol) accordingly to general procedure C; colorless solid; 70% (784 mg) yield after column chromatography (PhMe/EtOAc, 30/1 → 20/1); R_f_ = 0.48 (PhMe/EtOAc, 10/1); mp = 98 °C (*n*-heptane, CH_2_Cl_2_); [α]^20^_D_ = +54.5° (c = 1.0, CH_2_Cl_2_); ^1^H-NMR (400 MHz, CD_2_Cl_2_) δ = 8.19 (d, *J* = 8.3 Hz, 2H, H-Ar), 8.08 (d, *J* = 8.4 Hz, 2H, H-Ar), 7.75–7.97 (m, 12H, H-Ar), 7.64–7.74 (m, 2H, H-Ar), 7.54 (d, *J* = 6.7 Hz, 4H, H-Ar), 7.33–7.49 (m, 12H, H-Ar), 7.22–7.31 (m, 6H, H-Ar), 7.17–7.19 (m, 3H, H-Ar), 7.00 (t, *J* = 8.3 Hz, 1H, H-Ar), 5.96 (d, *J* = 8.3 Hz, 2H, H-Ar), 5.52 (d, *J* = 11.5 Hz, 2H, CH_2_Ar), 5.34 (d, *J* = 11.9 Hz, 2H, CH_2_Ar), 5.18–5.29 (m, 4H, CH_2_Ar), 5.13 (d, *J* = 11.7 Hz, 2H, CH_2_Ar), 5.02 (d, *J* = 11.9 Hz, 2H, CH_2_Ar), 4.71 (d, *J* = 3.4 Hz, 2H, H-1), 4.15–4.27 (m, 2H, H-3), 3.89–4.07 (m, 6H, H-4, H-5, H-6a), 3.76–3.88 (m, 4H, H-2, H-6b), 3.31 (s, 6H, CH_3_); ^13^C-NMR (101 MHz, CH_2_Cl_2_) δ = 158.8, 135.3, 134.5, 134.5, 134.3, 134.1, 132.3, 132.0, 132.0, 130.2, 129.5, 129.3, 128.9, 128.9, 128.9, 128.7, 128.6, 127.3, 127.2, 126.7, 126.6, 126.5, 126.5, 126.3, 126.1, 125.9, 125.8, 125.8, 124.8, 124.6, 124.4, 105.2 (C-Ar), 98.4 (C-1), 82.7 (C-3), 81.2 (C-2), 78.4 (CI), 77.9 (C-4), 73.9, 73.3, 71.9 (CH_2_Ar), 69.7 (C-5), 68.5 (C-6), 55.7 (CH_3_); HRMS (ESI-TOF) *m/z* [M + Na]^+^: calcd for C_86_H_77_IO_12_Na: 1451.43520, found: 1451.43439; Anal calcd for C_86_H_77_IO_12_: C 72.26, H 5.43, found: C 72.55, H 5.86.

## 4. Conclusions

In summary, thirteen new carbohydrate-based iodoarene catalysts were prepared. Three of them gave higher *er*’s in Kita’s spirolactonization than the previously reported ones. The way which led us to the improved catalyst design was described in detail. Investigation of the molecular structure and catalysts derived from different monosaccharides provided insights into the origin of the stereoselectivity of the carbohydrate-based iodoarene catalysts.

First, we changed the type of linkage between the carbohydrate moiety and the aromatic ring, and we found that a phenolic connection outmatched a benzylic linkage in terms of enantioselectivity of the catalyst. Next, we investigated if a different catalyst symmetry was beneficial for the catalyst design, and we found that a C_2_ symmetrical catalyst worked better than one with C_1_ symmetry. Afterwards, we introduced different carbohydrates as substituents as our catalyst. With d-galactose, the catalyst gave poor enantioselectivity by its application in Kita’s spirolactonization. The catalyst with d-galactose worked best. This led us to the assumption that position 4 is of major importance for the stereoselectivity. To gain further insight into the origin of stereoselectivity, we prepared crystals of one catalyst in order to obtain S-ray data to get the detailed molecular structure of our catalysts. With these findings at hand, we prepared improved catalysts with bulky substituents at position 4. One of our new catalysts gave a maximum *er* of 88:12 by its application in Kita’s spirolactonization.

## Figures and Tables

**Table 1:**
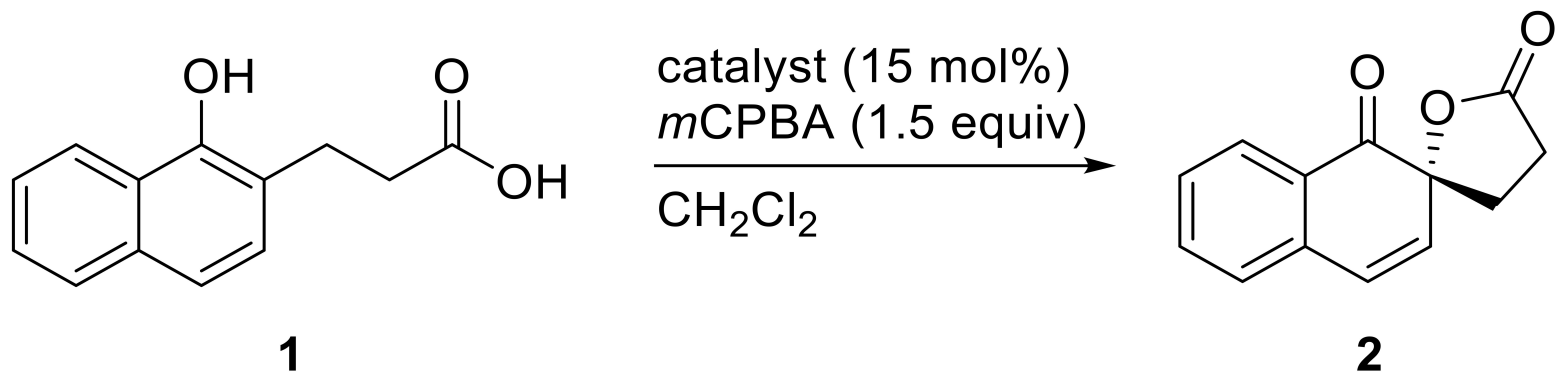
Application of catalysts in Kita’s oxidative spirolactonisation of 3-(1-hydroxynaphthalen-2-yl)propanoic acid.

Entry	Catalyst	Temperature	Yield^a^	*er* [R:S]^b^
1^c^	**8d**	−20 °C	77%	80:20
2	**11**	−20 °C	no conversion	n.d.^d^
3	**11**	rt	12%	55:45
4	**12**	−20 °C	no conversion	n.d. ^d^
5	**12**	rt	13%	60:40
6	**14**	−20 °C	no conversion	n.d. ^d^
7	**14**	rt	0% ^e^	n.d. ^d^
8	**15**	−20 °C	no conversion	n.d. ^d^
9	**15**	rt	0% ^e^	n.d. ^d^
10	**17**	−20 °C	no conversion	n.d. ^d^
11	**17**	rt	18%	52:48
12	**20**	−20 °C	81%	63:37
13	**34**	−20 °C	73%	70:30
14	**35**	−20 °C	67%	71:29
15	**36**	−20 °C	68%	55:45
16	**44**	−20 °C	72%	79:21
17	**45**	−20 °C	78%	81:19
18	**46**	−20 °C	74%	86:14
19	**50**	−20 °C	75%	88:12

^a^: Isolated yield. ^b^: Determined by chiral HPLC. ^c^: Taken from [20]. ^d^: Not determined. ^e^: No product was obtained; 3,4-dihydro-2*H*-naphtho [1,2-b]pyran-2-one was isolated instead.

## Supplementary Materials

Supplementary information (copies of NMR Spectra, HPLC chromatograms, and crystallographic data) is available at https://www.mdpi.com/1420-3049/24/21/3883/s1.
